# Association Between the Planetary Health Diet Index and Serum Uric Acid Levels and Incident Hyperuricemia: Findings From an Iranian Prospective Cohort Study

**DOI:** 10.1002/fsn3.72279

**Published:** 2026-08-23

**Authors:** Farzam Kamrani, Mobina Imannezhad, Elahe Etesami, Elham karimi, Matin Ghanavati, Jalaledin Mirzay Razaz, Reza Homayounfar, Ali Nikparast

**Affiliations:** ^1^ Department of Nutrition, Faculty of Medicine Mashhad University of Medical Sciences Mashhad Iran; ^2^ Department of Nutrition, SR.C. Islamic Azad University Tehran Iran; ^3^ Student Research Committee Tabriz University of Medical Sciences Tabriz Iran; ^4^ National Nutrition and Food Technology Research Institute (WHO Collaborating Center), Faculty of Nutrition Sciences and Food Technology Shahid Beheshti University of Medical Sciences Tehran Iran; ^5^ Department of Community Nutrition, Faculty of Nutrition and Food Technology, National Nutrition and Food Technology Research Institute Shahid Beheshti University of Medical Sciences Tehran Iran

**Keywords:** dietary patterns, EAT–Lancet diet, hyperuricemia, planetary health diet index, prospective cohort study, serum uric acid, sustainable diets

## Abstract

The EAT–Lancet planetary health diet aims to jointly promote human health and environmental sustainability, yet its relationship with uric acid metabolism remains largely unexplored. We prospectively examined the association between adherence to this dietary pattern and both incident hyperuricemia and serum uric acid levels, and tested whether insulin resistance mediated any observed link. Among 5773 Iranian adults free of hyperuricemia at baseline and followed for approximately five years, dietary intake was assessed using a validated food frequency questionnaire. Adherence to the EAT–Lancet diet was quantified with the Planetary Health Diet Index (PHDI; range 0–150). Hyperuricemia was defined as serum uric acid exceeding 7.0 mg/dL in men and 6.0 mg/dL in women. Multivariable logistic and linear regression models estimated associations, and bootstrapped causal mediation analysis quantified the indirect effect through HOMA‐IR. Incident hyperuricemia occurred in 1113 participants (cumulative incidence 19.3%). In the fully adjusted model, the highest versus lowest PHDI quartile was associated with 25% lower odds of hyperuricemia (OR 0.75; 95% CI 0.62–0.91; *p*‐trend < 0.001). Each SD increment in PHDI corresponded to a 0.053 mg/dL lower follow‐up serum uric acid after adjustment for baseline levels (*p* < 0.001). Mediation analysis suggested that HOMA‐IR accounted for a large proportion of the association (point estimate 91.0%; 95% CI 48.2%–232.5%), although the wide confidence interval calls for cautious interpretation; the direct effect was non‐significant. Findings were robust across sensitivity analyses, alternative EAT–Lancet scoring systems, and further adjustment for established dietary indices including the Mediterranean diet score, Healthy Eating Index 2015, Diet Quality Index Revised, and Dietary Approaches to Stop Hypertension score. Greater adherence to the planetary health diet was prospectively associated with lower serum uric acid and reduced hyperuricemia odds, a benefit that appeared to be mediated largely through insulin sensitivity.

## Graphical abstract

In 5773 adults followed for ~5 years, higher EAT–Lancet diet adherence was consistently associated with lower incident hyperuricemia and lower serum uric acid. Associations were observed across three EAT–Lancet scoring approaches and persisted after adjustment for established dietary‐quality indices. Baseline HOMA‐IR may partly mediate this association, with stronger associations observed in women, younger adults, normal‐weight participants, those with lower wealth, and those with higher physical activity.

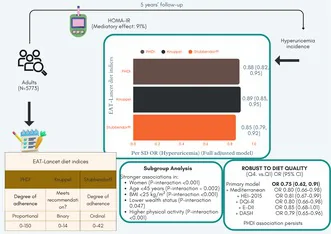

## 1 Introduction

Hyperuricemia, defined as serum uric acid > 7.0 mg/dL in men and > 6.0 mg/dL in women, is a key precursor of gout and has been linked to hypertension, chronic kidney disease, metabolic syndrome, and cardiovascular disease (Du et al. 2024). Worldwide prevalence has risen sharply in recent decades (Ngandeu‐Singwe et al. 2026), highlighting the role of modifiable factors such as diet.

Overall dietary patterns influence uric acid levels beyond single nutrients. Meta‐analyses show that plant‐based patterns are associated with lower hyperuricemia risk, while animal‐based and sweet patterns increase risk (Wen et al. 2024; Can et al. 2023). The proposed mechanisms underlying these associations include the uricosuric effect of vitamin C and folate, the inhibition of xanthine oxidase by flavonoids and isoflavones, and the binding of uric acid to dietary fiber in the gut, while the adverse effect of animal‐based patterns is thought to operate through purine loading, fructose‐driven ATP degradation in sweet dietary patterns, and the low‐grade systemic inflammation that accompanies diets rich in processed meats and saturated fats (Wen et al. 2024; Can et al. 2023).

The EAT‐Lancet Commission's reference diet, launched in 2019, set out to define a global eating pattern that could nourish a growing population without pushing Earth's systems past their breaking point (Willett et al. 2019). The framework establishes recommended intake ranges for a set of core food groups—emphasizing vegetables, fruits, whole grains, legumes, and nuts while setting firm upper limits on red meat, added sugars, and saturated fats (Willett et al. 2019). Translating these recommendations into a measurable score, however, has proven anything but straightforward. Researchers have developed several indices that differ considerably in how they handle food components, energy adjustment, and the scoring system itself—ranging from simple binary cut‐offs to more nuanced continuous scales—and these methodological choices directly affect how individuals are ranked and which associations with health outcomes emerge (Miranda et al. 2025).

Emerging evidence has linked the EAT–Lancet diet to lower risk of chronic kidney disease (Yang et al. 2026; Zhang et al. 2026; Ming et al. 2026), a condition intimately tied to urate handling because the kidneys are the primary route of uric acid excretion. Curiously, however, no prospective work has considered uric acid as an outcome. This is a striking omission given that serum urate is cleared primarily by the kidneys, that elevated concentrations predict both incident CKD and its progression, and that the very dietary exposures targeted by the EAT–Lancet framework—purine‐rich red meat and fructose from added sugars—are well‐established drivers of endogenous uric acid production (Johnson et al. 2023; Cheng et al. 2023). A recent cross‐sectional study of American adults by Tan et al. (2025) provided the first evidence linking the PHDI to lower odds of hyperuricemia, though the protective effect was weaker than that of conventional dietary indices and the cross‐sectional design precluded temporal inference.

Insulin resistance has been proposed as a plausible mediator linking diet to uric acid because insulin stimulates renal urate reabsorption through upregulation of URAT1 and other transporters in the proximal tubule, thereby reducing renal clearance of uric acid (Toyoki et al. 2017). We therefore selected HOMA‐IR as the primary mediator to test whether the EAT–Lancet diet might influence serum urate through improvements in insulin sensitivity.

This study therefore aimed to prospectively investigate the association between adherence to the original PHDI and both incident hyperuricemia and serum uric acid levels, and to identify the specific dietary components, population subgroups, and alternative EAT–Lancet indices that drive or modify these relationships.

## 2 Methods

This investigation was a prospective cohort study nested within the Monitoring of Metabolic Diseases Risk Factors in Tehran (MMRT) study—an ongoing, population‐based initiative designed to identify determinants of non‐communicable diseases among adults aged 20 to 70 years residing in Tehran, Iran (Emami et al. 2025). Between January 2017 and December 2018, a total of 8760 eligible individuals were invited to participate through a convenience sampling framework at a major urban referral health center. Of those invited, 7836 provided written informed consent and completed the baseline assessment, yielding a response rate of 89.4%. Each participant was assigned a unique anonymized identifier to ensure confidentiality. Baseline data were collected using standardized protocols, including structured face‐to‐face interviews, comprehensive clinical examinations, and laboratory analyses, covering sociodemographic characteristics, lifestyle behaviors, medical history, dietary habits, and anthropometric measures. Approximately five years later (January 2022 to December 2023), participants were re‐invited for a follow‐up assessment employing identical procedures. The follow‐up phase achieved an 86.7% retention rate, with 7600 individuals completing the reassessment.

**FIGURE 1:**
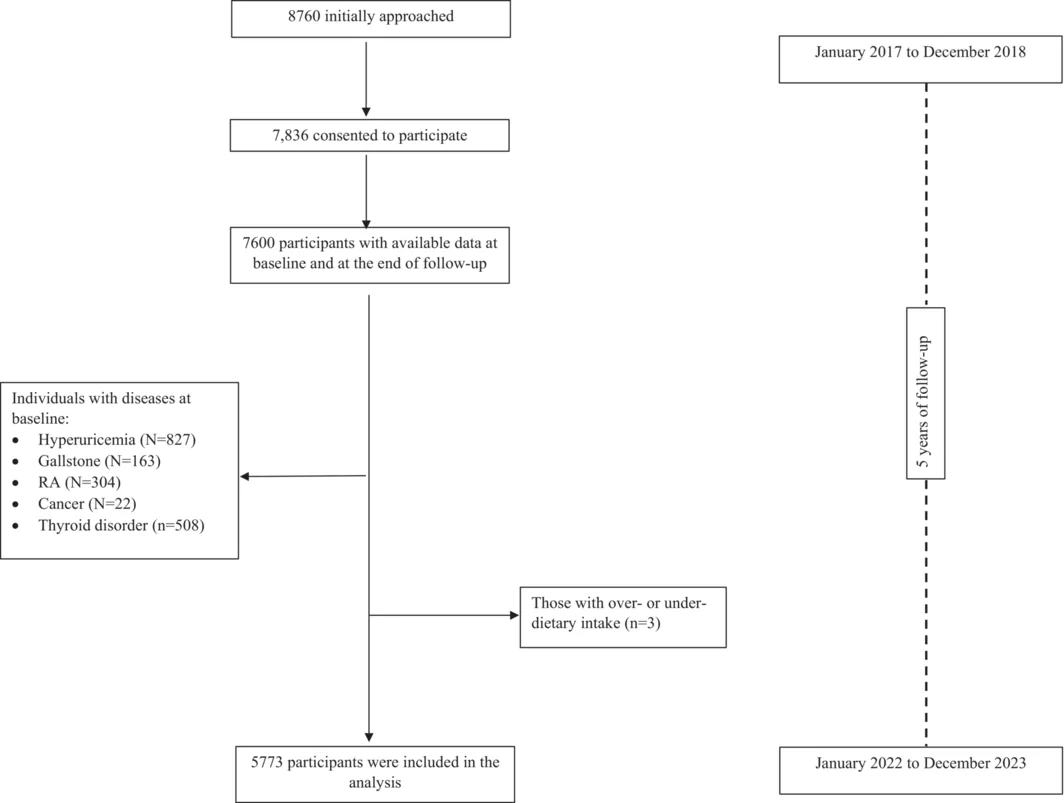
Flowchart of participant selection from initial approach to final analysis. From January 2017 to December 2018, 8760 individuals were initially approached, of whom 7836 consented to participate. After 5 years of follow‐up (January 2022 to December 2023), 7600 participants had available data at both baseline and the end of follow‐up. Exclusions at baseline included individuals with hyperuricemia (*n* = 827), gallstone (*n* = 163), rheumatoid arthritis (*n* = 304), cancer (*n* = 22), thyroid disorder (*n* = 508), and those with over‐ or under‐dietary intake (*n* = 3). The final analytic sample comprised 5773 participants.

For the present analysis, we applied a series of exclusion criteria to ensure the validity of the findings. We restricted the sample to participants who were free of hyperuricemia at baseline, had not reported a history of cancer, gallstone disease, rheumatic disorders, or thyroid conditions, and had plausible daily energy intakes (within the range of 1169 to 3227 kcal). After applying these criteria, the final analytical sample consisted of 5773 adults. A detailed flow diagram depicting the participant selection process is provided in Figure 1.

The study protocol was approved by the Institutional Review Board of Shahid Beheshti University of Medical Sciences (Ethics Code: IR.SBMU.NNFTRI.REC.1404.003). All procedures were conducted in strict accordance with the Declaration of Helsinki, and written informed consent was obtained from all participants prior to their inclusion.

### 2.1 Assessment of Non‐Dietary Covariates

Trained interviewers administered a standardized, pretested questionnaire to capture detailed information on demographics, socioeconomic status, and medical history, including age, sex, educational attainment, smoking history, prior diagnoses of chronic conditions, and current medication use.

Socioeconomic status was operationalized using a Wealth Score Index constructed through multiple correspondence analysis based on household asset ownership and service accessibility. The index incorporated indicators such as ownership of major household appliances (freezer, washing machine, dishwasher, vacuum cleaner), access to digital technologies (computer, internet, mobile phone, laptop), transportation assets (motorcycle or car, stratified by approximate market value), television type (standard or plasma), and history of international travel (Amini et al. 2022).

Habitual physical activity was quantified using the Modifiable Activity Questionnaire, a validated instrument for the Iranian population, with results expressed in metabolic equivalent task hours per week (Momenan et al. 2012). Hypertension was defined as systolic blood pressure ≥ 140 mmHg, diastolic blood pressure ≥ 90 mmHg, or current use of antihypertensive medication (Giles et al. 2009).

Anthropometric and body composition measurements were performed by trained health professionals following rigorous, standardized protocols. Body weight was recorded to the nearest 0.1 kg using a Seca 813 digital scale (Seca GmbH, Hamburg, Germany) with participants wearing light clothing and no shoes. Height was measured to the nearest 0.1 cm using a Seca 213 portable stadiometer. Body mass index was calculated as weight in kilograms divided by height in meters squared. Waist circumference was assessed at the midpoint between the lowest palpable rib and the iliac crest using a non‐elastic tape measure. Total body fat mass was quantified via dual‐energy X‐ray absorptiometry (Lunar iDXA, GE Healthcare, Wisconsin, USA), and the fat mass index was subsequently derived by dividing total fat mass in kilograms by height in meters squared, yielding a height‐normalized measure of adiposity (VanItallie et al. 1990).

Venous blood samples were collected after an 8‐ to 12‐h overnight fast. Fasting blood glucose, triglycerides, total cholesterol, and high‐density lipoprotein cholesterol were determined using enzymatic colorimetric assays on an automated analyzer (Selectra E, Vitalab, Holliston, The Netherlands) with commercial kits (Pars Azmoon, Iran). Low‐density lipoprotein cholesterol was calculated using the Friedewald formula. Fasting serum insulin was measured via electrochemiluminescence immunoassay, and insulin resistance was estimated using the Homeostatic Model Assessment of Insulin Resistance:

$$\text{HOMA}-\mathrm{IR}=\frac{\left[\text{fasting insulin}\;\left(\mathrm{\mu U}/\mathrm{mL}\right)\times \text{fasting glucose}\;\left(\mathrm{mg}/\mathrm{dL}\right)\right]}{405}$$

Hepatic enzymes—alanine aminotransferase, aspartate aminotransferase, and gamma‐glutamyl transferase—were analyzed using standard kinetic enzymatic methods. Serum creatinine was measured, and the estimated glomerular filtration rate (eGFR) was calculated using The Modification of Diet in Renal Disease (MDRD) criteria (Modification of Diet in Renal Disease Study Group 1992).

### 2.2 Dietary Intake Assessment and EAT‐Lancet Diet Adherence

Usual dietary intake over the preceding year was assessed using a 125‐item, semi‐quantitative Food Frequency Questionnaire (FFQ) adapted from the Willett questionnaire (Willett et al. 1985) and culturally tailored to capture prevalent items in the Iranian diet. In adapting the Willett FFQ, they removed items uncommon in the Iranian diet (e.g., bagels, cornbread) and incorporated traditional Iranian foods such as lavash bread, doogh (yogurt drink), and Persian stews (e.g., ghormeh sabzi). The final food list was refined through pilot 24‐h dietary recalls from the same source population and expert consensus from Iranian nutritionists (Eghtesad et al. 2023). At baseline, participants indicated their customary consumption frequency for each food item using prespecified categories (daily, weekly, monthly, or yearly). Portion sizes were primarily defined using standard serving sizes from the United States Department of Agriculture; for items not covered therein, common household measures were employed and subsequently converted to gram weights. Nutritional composition was derived primarily from the USDA nutrient database, supplemented by the Iranian Food Composition Table for traditional dishes absent from the USDA database.

Adherence to the EAT‐Lancet reference diet was evaluated using three distinct dietary indices to capture different operationalizations of the dietary pattern and assess the robustness of observed associations. The primary index was the Planetary Health Diet Index (PHDI), with the Knuppel EAT‐Lancet Score and the Stubbendorff EAT‐Lancet Index serving as complementary measures.

#### 2.2.1 Planetary Health Diet Index (PHDI)

The primary measure of adherence was the PHDI, originally developed and validated by Cacau et al. against nutrient profiles, overall dietary quality, and environmental impact indicators such as carbon footprint (Cacau et al. 2021). The index translates the EAT‐Lancet Commission's recommended intake ranges for major food groups within a 2500 kcal/day dietary pattern into a proportional scoring system. To ensure comparability across individuals with differing total energy intakes, all food group recommendations were operationalized using an energy‐density approach, whereby the caloric contribution of each food group was expressed as a percentage of total energy intake. Food items from the FFQ were aggregated into predefined food groups consistent with the EAT–Lancet classification; the specific items contributing to each PHDI component are detailed in Table S1.

The PHDI comprises 16 components organized into four conceptual domains. Adequacy components, for which higher intake reflects greater adherence, include nuts and peanuts, legumes, total fruits, total vegetables, and whole grains. Optimum components, for which moderate intake is preferred, encompass eggs, fish and seafood, tubers and potatoes, dairy products (excluding dairy fats), and vegetable oils. Two ratio components capture the proportion of dark green vegetables and red or orange vegetables relative to total vegetable intake. Moderation components, where lower intake indicates better adherence, consist of red meat (beef, lamb, pork), poultry and substitutes, animal fats, and added sugars. Each component is scored proportionally according to predefined cutoffs derived from the EAT‐Lancet reference diet energy contributions. Unlike binary scoring methods, the PHDI awards points gradually as consumption approaches or moves away from recommended ranges, thereby preserving inter‐individual variability and finer gradations of adherence (Cacau et al. 2021). Depending on the component, a maximum of 5 or 10 points is assigned. The total score ranges from 0 to 150, with higher values denoting stronger concordance with the EAT‐Lancet dietary pattern. Complete scoring criteria, with cut‐offs expressed as percentages of total energy intake, are provided in Table S2.

#### 2.2.2 Knuppel EAT‐Lancet Score (EPIC‐Oxford)

The Knuppel EAT‐Lancet Score was developed in the EPIC‐Oxford cohort to evaluate the association between adherence to the EAT‐Lancet Commission's dietary targets and major health outcomes (Knuppel et al. 2019). This index operationalizes the 14 key recommendations as binary components. For each component, participants receive a score of 1 if their energy‐adjusted intake—standardized to a 2500 kcal/day diet—falls within the recommended range, and 0 otherwise. The 14 components are then summed, yielding a possible total score of 0 to 14 points, with higher scores indicating greater adherence.

In the present study population, observed Knuppel scores ranged from 1 to 8, reflecting the binary nature of the scoring algorithm. Because the binary method produces integer values and ties are common at specific score levels, the data lacked sufficient distributional granularity to support quartile‐based categorization. Consequently, participants were classified into tertiles (*T*1–*T*3) for analyses employing this index. Notably, the interquartile range for all tertile groups was zero owing to the discrete integer scale; accordingly, the distribution is presented as median (minimum–maximum).

#### 2.2.3 Stubbendorff EAT‐Lancet Index

The Stubbendorff EAT‐Lancet Index was developed using the Malmö Diet and Cancer cohort and has been shown to predict all‐cause, cardiovascular, and cancer mortality (Stubbendorff et al. 2022). This index also comprises 14 food components aligned with the EAT‐Lancet reference diet targets. However, rather than employing binary scoring, each component is scored on a graduated scale from 0 to 3 points based on energy‐adjusted intake per 2500 kcal/day relative to predefined target intervals. This approach explicitly captures partial adherence: participants whose intake falls within the optimal range receive the maximum of 3 points, those with intakes moderately outside the range receive intermediate scores (1 or 2 points), and those with intakes substantially divergent from recommendations receive 0 points.

The 14 component scores are summed to produce a total ranging from 0 to 42 points, with higher scores reflecting closer alignment with the EAT‐Lancet dietary pattern. Compared with binary methods, the gradual scoring structure of the Stubbendorff index retains more distributional information and permits finer discrimination between levels of adherence within a population (Stubbendorff et al. 2022). Participants were categorized into quartiles based on their total index scores for the present analysis.

#### 2.2.4 Comparator Dietary Indices

To determine whether the association between PHDI adherence and hyperuricemia was independent of overall diet quality as captured by conventional dietary patterns, we additionally calculated five established dietary indices from the same FFQ data: the Mediterranean Diet Score (range 0–14) (Trichopoulou et al. 2003), the Healthy Eating Index 2015 (HEI‐2015; range 0–100) (Krebs‐Smith et al. 2018), the Diet Quality Index Revised (DQI‐R; range 0–100) (Haines et al. 1999), the Energy‐Adjusted Dietary Inflammatory Index (E‐DII) (Shivappa et al. 2014), and the Dietary Approaches to Stop Hypertension (DASH) score (range 8–40) (Fung et al. 2008). Each index was operationalized as a continuous variable (per standard deviation increment) in sensitivity analyses. Detailed scoring protocols, component definitions, food group assignments, and adaptations to the Iranian food composition database are provided in Table S7.

### 2.3 Ascertainment of Serum Uric Acid and Hyperuricemia

Fasting venous blood samples were collected at both baseline and follow‐up visits following an 8‐ to 12‐h overnight fast. Serum uric acid concentrations were determined using an enzymatic colorimetric method on an automated biochemical analyzer (Selectra E, Vitalab, Holliston, the Netherlands) with commercial reagent kits (Pars Azmoon, Iran). All assays were performed in the central laboratory of the study center under standardized quality control protocols to minimize inter‐batch variability.

Hyperuricemia was defined according to established clinical thresholds that account for sex‐specific physiological differences in uric acid metabolism. Specifically, hyperuricemia was diagnosed as a serum uric acid concentration exceeding 7.0 mg/dL in men and exceeding 6.0 mg/dL in women. This definition aligns with internationally recognized criteria and has been widely adopted in epidemiological studies investigating uric acid‐related outcomes (Ruiz‐García et al. 2024).

For the prospective analysis of incident hyperuricemia, the study sample was restricted to participants who were free of hyperuricemia at baseline. This analytic approach ensured that the observed associations between EAT‐Lancet diet adherence indices and hyperuricemia reflected new‐onset cases occurring during the approximately five‐year follow‐up period. Serum uric acid concentration at follow‐up was additionally analyzed as a continuous outcome variable, expressed in mg/dL, with adjustment for baseline serum uric acid to evaluate the relationship between dietary adherence and change in uric acid levels over time.

### 2.4 Statistical Analysis

Baseline characteristics of the study population and their dietary intakes were summarized across quartiles of the PHDI. Continuous variables are presented as means ± standard deviations, and categorical variables are reported as frequencies with percentages. Linear trends across PHDI quartiles were evaluated by assigning the median value of each quartile and modeling it as a continuous variable in linear regression for continuous characteristics; for categorical variables, the Mantel–Haenszel *χ* ^2^ test was employed.

The primary analyses used multivariable logistic regression to estimate odds ratios and 95% confidence intervals for incident hyperuricemia, and multivariable linear regression to examine follow‐up serum uric acid concentration adjusted for baseline levels. The PHDI was operationalized both as a categorical variable (quartiles, with the lowest quartile serving as the reference) and as a continuous variable (per standard deviation increment) to assess the dose–response nature of the associations. A sequential adjustment strategy was implemented across three models. Model 1 was adjusted for age and sex. Model 2 additionally included body mass index, physical activity, Wealth Score Index, educational level, smoking status, and hypertension. The fully adjusted model (Model 3) further accounted for total dietary energy intake.

To explore the potential non‐linearity of the dose–response relationship and to visually depict the functional form of the association, a multivariable‐adjusted restricted cubic spline model with three knots, placed at the 10th, 50th, and 90th percentiles of the PHDI, was fitted. This analysis was adjusted for all covariates included in Model 3.

To formally dissect the pathways underpinning the PHDI–hyperuricemia link, we conducted a causal mediation analysis to determine whether insulin resistance (measured by HOMA‐IR at baseline) serves as a statistical mediator. Analyses were implemented in R (version 4.5.2) using the mediation package, with mediator and outcome models specified using the same covariates as in the primary multivariable analysis. Nonparametric bootstrapping with 10,000 resamples was used to derive stable estimates for the indirect (mediated), direct, and total effects, along with their 95% confidence intervals. The proportion mediated was calculated, and its 95% confidence interval was obtained from the bootstrap distribution.

Pre‐specified stratified analyses were conducted to explore potential effect modification by age (< 45 vs. ≥ 45 years), sex, body mass index (< 25 vs. ≥ 25 kg/m^2^), smoking status, socioeconomic status (Wealth Score Index below vs. above the mean), and physical activity level (below vs. above the mean). In these subgroup analyses, the stratifying variable was included as a main effect along with a product interaction term, and the corresponding continuous covariate was omitted from the adjustment set for that specific subgroup to avoid over‐adjustment. Statistical evidence of interaction was assessed using the *p*‐value for the interaction term.

The robustness of the primary findings was evaluated through a series of sensitivity analyses. To determine whether the observed associations were independent of specific metabolic pathways, additional models were constructed by further adjusting Model 3 for baseline HOMA‐IR, C‐reactive protein, the triglyceride‐to‐high‐density lipoprotein cholesterol ratio, serum creatinine, and dietary protein intake expressed as a percentage of total energy. To evaluate whether the association was robust to the exclusion of participants with pre‐existing kidney impairment or gout, we repeated the primary analysis after removing individuals with baseline eGFR < 60 mL/min/1.73 m^2^. To ascertain which dietary components drove the overall association, the relationship between each of the 16 individual PHDI components (per standard deviation increment) and incident hyperuricemia was examined using multivariable logistic regression with full covariate adjustment.

To evaluate whether the observed association between PHDI adherence and hyperuricemia was independent of overall diet quality rather than merely reflecting adherence to any high‐quality dietary pattern, we conducted an additional set of sensitivity analyses in which Model 3 was further adjusted—one at a time—for each of the five comparator dietary indices (Mediterranean Diet Score, HEI‐2015, DQI‐R, E‐DII, and DASH score), each entered as a continuous variable per standard deviation increment. This mutual adjustment approach tested whether the PHDI–hyperuricemia association persisted after accounting for shared variance with established dietary quality constructs.

Finally, to assess whether the findings were contingent on the specific scoring system employed, all primary analyses were repeated using two alternative EAT‐Lancet adherence metrics: The Knuppel EAT‐Lancet Score (analyzed in tertiles owing to its restricted distributional range) and the Stubbendorff EAT‐Lancet Index (analyzed in quartiles).

All statistical tests were two‐tailed with a significance threshold set at 0.05, and analyses included only participants with complete data for all examined variables. Data analysis was carried out using IBM SPSS Statistics software version 26.0 (IBM Corp., Armonk, NY, USA) and R statistical software version 4.5.0 (R Foundation for Statistical Computing, Vienna, Austria).

## 3 Results

### 3.1 Participant Characteristics

The final analytical sample comprised 5773 participants (mean age 42.5 ± 10.1 years; 50.9% men). Table 1 presents the baseline characteristics of study participants across quartiles of the PHDI. The mean PHDI score was 50.7 ± 5.8 points. Participants in the highest quartile of adherence, compared with those in the lowest quartile, tended to be younger, had a lower body mass index (27.7 ± 4.5 vs. 28.5 ± 4.6 kg/m^2^; *p*‐trend = 0.001), smaller waist circumference (86.2 ± 15.2 vs. 90.8 ± 16.5 cm; *p*‐trend = 0.001), and lower fat mass index (7.8 ± 3.8 vs. 9.1 ± 3.9 kg/m^2^; *p*‐trend = 0.001). They also exhibited more favorable cardiometabolic profiles, including lower systolic blood pressure, fasting blood glucose, fasting insulin, HOMA‐IR, triglycerides, total cholesterol, LDL‐C, ALT, AST, GGT, and CRP (all *p*‐trend ≤ 0.001). eGFR was higher across ascending PHDI quartiles (*p*‐trend < 0.001). The prevalence of hypertension, non‐alcoholic fatty liver disease, Type 2 diabetes mellitus, and chronic kidney disease decreased across increasing quartiles of PHDI adherence (all *p*‐trend < 0.05). Mean serum uric acid concentration at baseline declined from 4.79 ± 1.05 mg/dL in the lowest quartile to 4.62 ± 1.03 mg/dL in the highest quartile (*p*‐trend < 0.001).

**TABLE 1:** Baseline characteristics of participants across quartiles of Planetary Health Diet Index.^a^

Characteristics	Total population (*N* = 5773)	Quartile 1 (*N* = 1385)	Quartile 2 (*N* = 1441)	Quartile 3 (*N* = 1493)	Quartile 4 (*N* = 1454)	*p*‐trend^b^
Age	42.52 ± 10.05	43.54 ± 10.01	43.02 ± 10.60	41.87 ± 9.65	41.71 ± 9.84	**< 0.001**
Men, *n* (%)	2938 (50.9%)	642 (46.4%)	792 (55.0%)	808 (54.1%)	696 (47.9%)	**< 0.001**
Smoker, *n* (%)	670 (11.6%)	149 (10.8%)	189 (13.1%)	167 (11.2%)	165 (11.3%)	0.272
Education, *n* (%)						0.305
Low	1062 (18.4%)	265 (19.1%)	278 (19.3%)	272 (18.2%)	247 (17.0%)	
Middle	3404 (59.0%)	784 (56.6%)	845 (58.6%)	897 (60.1%)	878 (60.4%)	
High	1307 (22.6%)	336 (24.3%)	318 (22.1%)	324 (21.7%)	329 (22.6%)	
WSI	70.46 ± 14.10	68.54 ± 13.75	70.08 ± 14.46	72.34 ± 13.93	70.72 ± 14.01	**< 0.001**
Physical activity (MET/h/week)	23.72 ± 2.75	23.23 ± 2.56	23.61 ± 2.76	24.11 ± 2.82	23.92 ± 2.78	**< 0.001**
Weight (kg)	87.12 ± 13.99	89.85 ± 14.29	88.65 ± 13.82	84.05 ± 13.49	86.16 ± 13.66	**< 0.001**
Height (cm)	166.66 ± 6.14	166.62 ± 6.20	166.92 ± 6.11	166.71 ± 6.11	166.38 ± 6.15	0.196
Waist circumference (cm)	87.03 ± 15.65	90.81 ± 16.45	87.56 ± 15.99	83.82 ± 14.20	86.22 ± 15.16	**0.001**
Body mass index (kg/m^**2**^)	27.89 ± 4.59	28.45 ± 4.57	28.34 ± 4.62	27.09 ± 4.50	27.74 ± 4.54	**0.001**
Fat mass index (kg/m^**2**^)	8.02 ± 3.81	9.10 ± 3.94	8.23 ± 3.84	7.06 ± 3.40	7.77 ± 3.76	**0.001**
SBP (mmHg)	118.46 ± 7.86	119.07 ± 8.00	118.94 ± 8.01	117.81 ± 7.39	118.09 ± 7.96	**0.001**
DBP (mmHg)	72.89 ± 9.30	73.38 ± 9.50	72.80 ± 9.15	72.53 ± 9.31	72.89 ± 9.24	0.117
Fasting blood sugar (mg/dL)	98.84 ± 14.86	100.00 ± 14.84	99.50 ± 14.73	97.14 ± 15.00	98.83 ± 14.73	**0.001**
Fasting Insulin (μU/mL)	8.99 ± 3.05	10.00 ± 3.14	9.21 ± 3.19	8.21 ± 2.72	8.61 ± 2.84	**0.001**
HOMA‐IR	1.43 ± 0.61	1.67 ± 0.62	1.50 ± 0.61	1.22 ± 0.54	1.34 ± 0.58	**0.001**
Triglyceride (mg/dL)	139.33 ± 16.93	141.95 ± 17.44	140.39 ± 17.44	136.67 ± 15.89	138.53 ± 16.53	**0.001**
Total cholesterol (mg/dL)	172.03 ± 27.34	174.86 ± 29.05	173.82 ± 27.21	169.31 ± 25.62	170.34 ± 27.13	**0.001**
LDL‐C (mg/dL)	95.70 ± 15.36	97.33 ± 15.05	96.41 ± 15.33	93.90 ± 15.00	95.27 ± 15.85	**0.001**
HDL‐C (mg/dL)	43.89 ± 7.02	42.55 ± 6.79	43.51 ± 7.18	44.86 ± 6.97	44.52 ± 6.89	**0.001**
ALT (U/L)	27.38 ± 4.78	28.21 ± 4.85	27.96 ± 4.74	26.64 ± 4.57	26.77 ± 4.77	**0.001**
AST (U/L)	24.94 ± 4.99	25.69 ± 5.11	25.51 ± 4.86	24.24 ± 4.86	24.37 ± 4.98	**0.001**
GGT (U/L)	26.87 ± 9.37	30.64 ± 9.04	27.95 ± 9.36	23.56 ± 8.62	25.62 ± 8.97	**0.001**
CRP (mg/L)	2.48 ± 2.18	3.31 ± 2.41	2.59 ± 2.21	1.84 ± 1.76	2.24 ± 2.06	**0.001**
Serum uric acid (mg/dL)	4.71 ± 1.04	4.79 ± 1.05	4.77 ± 1.06	4.65 ± 1.01	4.62 ± 1.03	**0.001**
eGFR (mL/min/1.73 m^**2**^)	89.97 ± 16.23	87.52 ± 16.36	90.27 ± 16.84	91.69 ± 15.22	90.25 ± 16.26	**0.001**
Hypertension, *n* (%)	1153 (20.0%)	307 (22.2%)	296 (20.5%)	265 (17.7%)	285 (19.6%)	**0.027**
Non‐alcoholic fatty liver disease	1099 (19.0%)	420 (30.3%)	293 (20.3%)	158 (10.6%)	228 (15.7%)	**< 0.001**
Type 2 diabetes mellitus, *n* (%)	532 (9.2%)	171 (12.3%)	161 (11.2%)	94 (6.3%)	106 (7.3%)	**< 0.001**
Chronic kidney disease (< 60 mL/min/1.73 m^**2**^), *n* (%)	83 (1.4%)	29 (2.1%)	25 (1.7%)	14 (0.9%)	15 (1.0%)	**0.024**
Planetary health diet score	50.66 ± 5.75	43.72 ± 2.46	48.70 ± 1.10	51.57 ± 0.46	58.26 ± 3.99	**0.001**

Abbreviations: ALT, alanine aminotransferase; AST, aspartate aminotransferase; CRP, C‐reactive protein; DBP, diastolic blood pressure; GGT, gamma‐glutamyl transferase; HDL‐C, high density lipoprotein cholesterol; HOMA‐IR, Homeostatic Model Assessment for Insulin Resistance; LDL‐C, low density lipoprotein cholesterol; SBP, systolic blood pressure; WSI, Wealth Score Index.

^a^ Data are presented as mean ± SD for continuous variables and number (percent) for categorical variables.

^b^ *p*‐trend was obtained from linear regression analysis for continuous variables and *χ* ^2^ test for categorical variables, respectively, according to the category of EAT‐Lancet diet.

### 3.2 Dietary Intake Patterns

Dietary composition across quartiles of the PHDI is detailed in Table 2. Total energy intake was modestly lower in the highest compared with the lowest quartile (2155.7 ± 350.9 vs. 2278.3 ± 367.4 kcal/day; *p*‐trend < 0.001). As adherence to the PHDI increased, participants consumed a greater proportion of energy from carbohydrates and protein, and a lower proportion from fat (all *p*‐trend < 0.001). Consumption of whole grains, legumes, fruits, vegetables, nuts, low‐fat dairy, and cheese increased significantly across quartiles (all *p*‐trend < 0.001), whereas intakes of refined grains, red meat, processed meat, animal fats, vegetable oils, and added sugars decreased (all *p*‐trend < 0.001). Dietary fiber, folate, vitamin C, calcium, magnesium, potassium, and iron intakes rose with greater adherence, while saturated fatty acids, trans fatty acids, MUFA, and PUFA intakes declined (all *p*‐trend < 0.001). Fish consumption was highest in the third quartile and lowest in the fourth quartile (*p*‐trend < 0.001).

**TABLE 2:** Dietary intake of participants across quartiles of Planetary Health Diet Index.^a^

	Quartile 1	Quartile 2	Quartile 3	Quartile 4	*p*‐trend^b^
Total energy intake (kcal)	2278.32 ± 367.35	2281.07 ± 343.90	2205.38 ± 324.16	2155.68 ± 350.91	**< 0.001**
Carbohydrate intake (% of energy)	60.26 ± 3.72	60.37 ± 3.57	60.90 ± 3.46	61.20 ± 3.87	**< 0.001**
Protein intake (% of energy)	12.67 ± 1.36	12.99 ± 1.36	13.56 ± 1.29	13.56 ± 1.42	**< 0.001**
Fat intake (% of energy)	29.08 ± 4.10	28.68 ± 3.86	27.68 ± 3.71	27.37 ± 4.15	**< 0.001**
Whole grain (g/day)	10.68 ± 7.34	17.29 ± 6.77	26.58 ± 9.27	23.07 ± 9.79	**< 0.001**
Refined Grain (g/day)	397.52 ± 78.67	389.64 ± 74.31	369.95 ± 68.88	375.80 ± 73.24	**< 0.001**
Legumes (g/day)	55.21 ± 18.80	60.59 ± 20.69	67.34 ± 19.69	63.09 ± 19.94	**< 0.001**
Fruits (g/day)	332.80 ± 65.20	345.25 ± 63.90	359.40 ± 63.71	351.12 ± 62.19	**< 0.001**
Vegetables (g/day)	471.65 ± 101.51	485.15 ± 100.02	516.01 ± 102.66	502.23 ± 102.94	**< 0.001**
Nuts (g/day)	3.78 ± 3.88	5.40 ± 4.22	5.82 ± 3.93	5.46 ± 3.89	**< 0.001**
Fish (g/day)	9.11 ± 4.85	8.97 ± 5.35	9.70 ± 5.72	6.84 ± 5.81	**< 0.001**
Eggs (g/day)	29.36 ± 13.22	31.01 ± 13.51	30.66 ± 13.61	29.61 ± 14.18	0.844
Dairy Low‐Fat (g/day)	94.58 ± 27.72	100.18 ± 29.43	103.45 ± 29.42	100.66 ± 28.56	**< 0.001**
Cheese (g/day)	22.29 ± 9.40	23.39 ± 9.49	25.58 ± 8.67	24.83 ± 9.41	**< 0.001**
Vegetable oils (g/day)	7.16 ± 4.90	6.78 ± 5.21	6.35 ± 5.05	4.40 ± 4.94	**< 0.001**
Animal fats (g/day)	22.75 ± 13.40	20.68 ± 12.38	16.72 ± 11.27	16.79 ± 12.95	**< 0.001**
Chicken (g/day)	31.34 ± 12.89	33.29 ± 13.53	34.85 ± 13.64	33.63 ± 13.54	**< 0.001**
Red Meat (g/day)	18.14 ± 8.91	18.03 ± 8.72	16.30 ± 8.74	16.44 ± 9.05	**< 0.001**
Processed Meat (g/day)	9.33 ± 7.22	8.73 ± 6.53	7.74 ± 6.14	8.27 ± 6.59	**< 0.001**
Added Sugar (g/day)	50.18 ± 22.89	48.64 ± 23.01	42.72 ± 21.35	38.19 ± 25.44	**< 0.001**
Sugary Drinks (g/day)	87.31 ± 51.20	81.41 ± 50.80	67.42 ± 42.18	71.38 ± 46.93	0.237
Fruit Juice (g/day)	8.41 ± 11.12	8.39 ± 11.47	7.89 ± 10.90	8.06 ± 11.00	**< 0.001**
Coffee (g/day)	3.17 ± 2.67	3.24 ± 2.57	3.18 ± 2.59	3.30 ± 2.63	0.298
Tea (g/day)	971.14 ± 636.18	1003.19 ± 630.45	932.72 ± 604.16	977.80 ± 608.58	0.477
Dietary Fiber (g/day)	24.47 ± 3.36	25.59 ± 3.41	26.88 ± 3.35	26.05 ± 3.38	**< 0.001**
Saturated fatty acid (g/day)	27.12 ± 6.56	26.57 ± 5.93	24.79 ± 5.56	24.43 ± 6.26	**< 0.001**
Trans fatty acid (g/day)	0.68 ± 0.38	0.64 ± 0.37	0.53 ± 0.33	0.57 ± 0.37	**< 0.001**
MUFA (g/day)	23.09 ± 5.91	22.53 ± 5.44	20.83 ± 5.17	19.76 ± 5.51	**< 0.001**
PUFA (g/day)	12.17 ± 3.41	12.46 ± 3.36	12.05 ± 3.26	11.10 ± 3.16	**< 0.001**
ALA (18:3 n‐3) (g/day)	0.65 ± 0.29	0.64 ± 0.29	0.59 ± 0.28	0.53 ± 0.26	**< 0.001**
EPA (20:5 n‐3) (g/day)	0.015 ± 0.004	0.015 ± 0.004	0.016 ± 0.004	0.014 ± 0.004	**0.044**
DHA (22:6 n‐3) (g/day)	0.040 ± 0.012	0.042 ± 0.013	0.044 ± 0.013	0.038 ± 0.013	**0.007**
Vitamin D (IU/day)	57.21 ± 17.62	58.16 ± 18.33	59.38 ± 18.74	51.56 ± 18.72	**< 0.001**
Vitamin C (mg/day)	129.43 ± 27.48	133.13 ± 28.49	139.69 ± 27.56	135.47 ± 27.19	**< 0.001**
Vitamin E (mg/day)	8.29 ± 1.70	8.53 ± 1.78	8.38 ± 1.73	7.87 ± 1.68	**< 0.001**
Vitamin B12 (mcg)	8.01 ± 3.50	8.04 ± 3.51	7.93 ± 3.49	7.79 ± 3.32	0.063
Folate (μg/day)	517.72 ± 79.11	537.21 ± 80.63	551.59 ± 78.35	543.16 ± 79.51	**< 0.001**
Niacin (mg/day)	20.88 ± 3.57	21.42 ± 3.53	21.58 ± 3.43	21.08 ± 3.50	0.079
Choline (mg/day)	294.62 ± 54.11	305.01 ± 54.41	307.77 ± 54.97	298.92 ± 56.20	**0.020**
Calcium (mg/day)	838.11 ± 109.55	857.47 ± 106.71	866.70 ± 108.69	855.43 ± 107.93	**< 0.001**
Magnesium (mg/day)	305.77 ± 38.69	314.67 ± 38.55	318.46 ± 37.33	313.95 ± 38.55	**< 0.001**
Potassium (mg/day)	3508.33 ± 479.04	3589.96 ± 458.93	3639.55 ± 445.38	3574.18 ± 460.81	**< 0.001**
Sodium (mg/day)	3249.20 ± 933.04	3155.24 ± 862.78	2987.56 ± 801.10	3005.53 ± 866.08	**< 0.001**
Zinc (mg/day)	9.49 ± 1.45	9.71 ± 1.43	9.67 ± 1.41	9.56 ± 1.46	0.347
Iron (mg/day)	14.46 ± 2.06	14.89 ± 2.03	15.21 ± 2.01	14.93 ± 2.04	**< 0.001**
Selenium (μg/day)	90.93 ± 17.46	93.80 ± 17.36	94.43 ± 17.16	91.62 ± 17.02	0.212

^a^ Data are presented as mean ± standard error for continuous variables and number (percent) for non‐continuous variables.

^b^ *p*‐trend was obtained from linear regression analysis according to the category of EAT‐Lancet diet.

### 3.3 PHDI and Hyperuricemia Incidence

Over the approximately five‐year follow‐up period, 1113 incident cases of hyperuricemia were documented among the 5773 participants who were free of the condition at baseline, corresponding to a cumulative incidence of 19.3%. Table 3 shows the odds ratios for hyperuricemia incidence according to PHDI adherence. In the crude model, participants in the highest quartile of the PHDI had 46% lower odds of developing hyperuricemia compared with those in the lowest quartile (OR: 0.54; 95% CI: 0.45, 0.64; *p*‐trend < 0.001). This inverse association was attenuated but remained statistically significant after sequential adjustment for demographic, lifestyle, and dietary covariates. In the fully adjusted model (Model 3), the ORs across increasing quartiles were 1.00 (reference), 0.81 (95% CI: 0.68, 0.98), 0.62 (95% CI: 0.51, 0.76), and 0.75 (95% CI: 0.62, 0.91), respectively (*p*‐trend < 0.001). Each standard deviation increment in the PHDI score was associated with a 12% reduction in hyperuricemia odds (OR: 0.88; 95% CI: 0.82, 0.95; *p* = 0.001).

**TABLE 3:** Odds ratios (95% confidence intervals) for hyperuricemia incidence according to the proportion of Planetary Health Diet Index.

	Q1	Q2	Q3	Q4	*p*‐trend	Continues (per SD increment)	*p*
Median (IQR)	44.22 (2.98)	48.70 (1.86)	51.58 (0.64)	57.79 (5.73)			
Cases/populations	372/1385	304/1441	198/1493	239/1454			
Crude model	1.00 (Ref)	**0.73 (0.61, 0.87)**	**0.42 (0.34, 0.50)**	**0.54 (0.45, 0.64)**	< 0.001	**0.76 (0.71, 0.81)**	< 0.001
Model 1^a^	1.00 (Ref)	**0.74 (0.62, 0.88)**	**0.43 (0.36, 0.52)**	**0.56 (0.46, 0.67)**	< 0.001	**0.77 (0.72, 0.83)**	< 0.001
Model 2^b^	1.00 (Ref)	0.77 (0.65, 0.93)	**0.54 (0.44, 0.65)**	**0.64 (0.53, 0.77)**	< 0.001	**0.83 (0.77, 0.89)**	< 0.001
Model 3^c^	1.00 (Ref)	**0.81 (0.68, 0.98)**	**0.62 (0.51, 0.76)**	**0.75 (0.62, 0.91)**	< 0.001	**0.88 (0.82, 0.95)**	0.001

*Note:* Significant *p*‐values are highlighted in bold.

^a^ Model 1 adjusted for age and sex.

^b^ Model 2 additionally adjusted for Model 1, body mass index, physical activity, wealth score index, education level, smoking, and hypertension.

^c^ Model 3 additionally adjusted for Model 2 and dietary energy intake.

**FIGURE 2:**
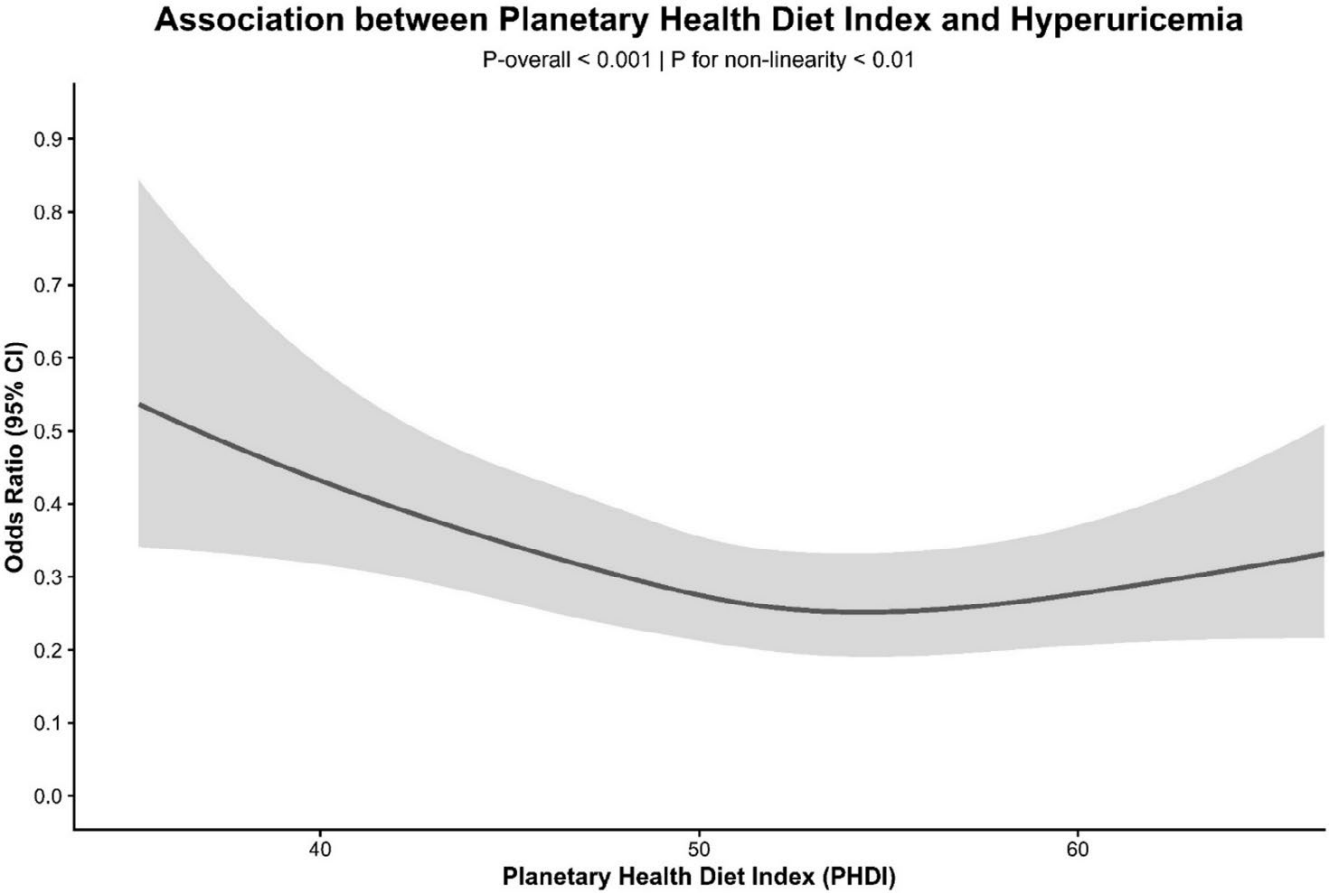
Dose–response relationship between adherence to the Planetary Health Diet Index and incident hyperuricemia. The solid line represents the adjusted odds ratio, and the shaded area indicates the 95% confidence interval. The reference value is set at the 10th percentile of the PHDI score. The model is adjusted for age, sex, total energy intake, physical activity, smoking status, alcohol consumption, education level, body mass index, and baseline estimated glomerular filtration rate. Histogram bars at the bottom of the figure represent the distribution of the study population across the PHDI continuum. CI, confidence interval; PHDI, Planetary Health Diet Index.

In the fully adjusted restricted cubic spline model, we observed a sharp, non‐linear inverse association between the PHDI and incident hyperuricemia (*p*‐overall < 0.001, *p*‐nonlinearity = 0.01) (Figure 2). The curve plateaued beyond a score of approximately 50, suggesting the full protective association was largely realized at moderate adherence levels.

### 3.4 Serum Uric Acid Levels

Consistent with the categorical findings, linear regression analyses (Table 4) revealed a significant inverse association between the continuous PHDI score and serum uric acid concentrations. In the fully adjusted model, each SD increase in PHDI score was associated with a 0.053 mg/dL lower follow‐up serum uric acid (*B* = −0.053; *p* < 0.001).

**TABLE 4:** Unstandardized coefficients for serum uric acid levels according to each SD increase of the Planetary Health Diet Index score.

	*B* (95% CI)	*p*
Crude + baseline serum uric acid	**−0.119 (−0.150, −0.089)**	< 0.001
Model 1^a^	**−0.116 (−0.146, −0.087)**	< 0.001
Model 2^b^	**−0.089 (−0.118, −0.060)**	< 0.001
Model 3^c^	**−0.053 (−0.082, −0.023)**	< 0.001

*Note:* All models were adjusted for baseline serum uric acid concentration. Significant *p*‐values are highlighted in bold.

^a^ Model 1 adjusted for age and sex.

^b^ Model 2 additionally adjusted for Model 1, body mass index, physical activity, wealth score index, education level, smoking, and hypertension.

^c^ Model 3 additionally adjusted for Model 2 and dietary energy intake.

### 3.5 Mediation by Insulin Resistance

**FIGURE 3:**
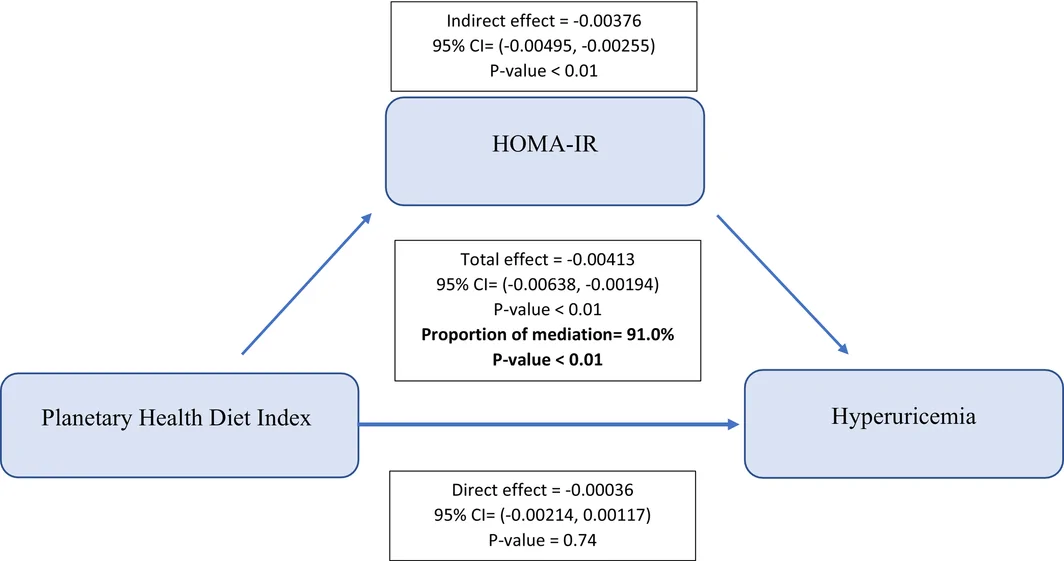
Mediation analysis of HOMA‐IR on the relationship between Planetary Health Diet Index and hyperuricemia. HOMA‐IR was measured at baseline. The total effect of the Planetary Health Diet Index on hyperuricemia was −0.00413 (95% CI: −0.00638 to −0.00194; *p* < 0.01). The indirect effect through HOMA‐IR was −0.00376 (95% CI: −0.00495 to −0.00255; *p* < 0.01), and the direct effect was −0.00036 (95% CI: −0.00214 to 0.00117; *p* = 0.74). The proportion of mediation was 91.0% (*p* < 0.01), indicating that HOMA‐IR almost fully mediated the observed association.

Given the attenuation observed when HOMA‐IR was added to the fully adjusted model, we formally tested whether insulin resistance functioned as a mediator on the causal pathway linking PHDI adherence to incident hyperuricemia (Figure 3). The total effect of the PHDI on hyperuricemia risk was statistically significant (−0.00413; 95% CI: −0.00638 to −0.00194; *p* < 0.01). Decomposition of this total effect revealed that the indirect pathway operating through HOMA‐IR carried nearly the entire signal, with an indirect effect of −0.00376 (95% CI: −0.00495 to −0.00255; *p* < 0.01). In contrast, the direct effect of the PHDI on hyperuricemia—the portion not routed through insulin resistance—was negligible and non‐significant (−0.00036; 95% CI: −0.00214 to 0.00117; *p* = 0.74). The point estimate for the proportion mediated was 91.0%; however, the 95% bootstrap confidence interval was wide (48.2% to 232.5%), indicating considerable uncertainty and the possibility of inconsistent mediation. Therefore, while the analysis suggests that insulin resistance accounts for a large share of the observed association, the exact proportion should be interpreted cautiously.

### 3.6 Subgroup Analyses

Table 5 presents the results of subgroup analyses stratified by potential effect modifiers. The inverse association between PHDI adherence and hyperuricemia incidence was more pronounced among participants younger than 45 years (OR for Q4 vs. Q1: 0.69; 95% CI: 0.53, 0.91; *p*‐trend = 0.001) compared with those aged 45 years and older (OR: 0.82; 95% CI: 0.62, 1.08; *p*‐trend = 0.074; *p* for interaction = 0.002). A stronger protective association was observed in women (OR for Q4 vs. Q1: 0.68; 95% CI: 0.52, 0.88; *p*‐trend < 0.001) than in men (OR: 0.83; 95% CI: 0.63, 1.10; *p*‐trend = 0.085; *p* for interaction < 0.001). The protective association was evident among participants with BMI ≥ 25 kg/m^2^ (OR for Q4 vs. Q1: 0.75; 95% CI: 0.60, 0.92; *p*‐trend = 0.001), but did not reach statistical significance in those with BMI < 25 kg/m^2^ (*p*‐trend = 0.182; *p* for interaction < 0.001). The inverse association was significant among those with lower wealth score index (*p*‐trend = 0.001) and among non‐smokers (*p*‐trend < 0.001), with significant interaction by wealth status (*p* for interaction = 0.047). The protective association was observed across both low and high physical activity strata, though the pattern differed (*p* for interaction < 0.001).

**TABLE 5:** Subgroup analysis of the association between Planetary Health Diet Index and the odds of hyperuricemia incidence.

	Q1	Q2	Q3	Q4	*p*‐trend	*p* for interaction
Age						
< 45 years old	1.00 (Ref)	0.85 (0.65, 1.09)	**0.58 (0.44, 0.77)**	**0.69 (0.53, 0.91)**	**0.001**	**0.002**
> 45 years old	1.00 (Ref)	0.80 (0.62, 1.04)	**0.67 (0.50, 0.90)**	0.82 (0.62, 1.08)	0.074	
Sex						
Men	1.00 (Ref)	0.83 (0.64, 1.08)	**0.68 (0.51, 0.90)**	0.83 (0.63, 1.10)	0.085	**< 0.001**
Women	1.00 (Ref)	0.83 (0.64, 1.08)	**0.58 (0.43, 0.77)**	**0.68 (0.52, 0.88)**	**< 0.001**	
Body mass index						
< 25 kg/m^2^	1.00 (Ref)	0.71 (0.44, 1.15)	**0.56 (0.35, 0.90)**	0.78 (0.48, 1.26)	0.182	**< 0.001**
≥ 25 kg/m^2^	1.00 (Ref)	0.86 (0.71, 1.05)	**0.63 (0.51, 0.79)**	**0.75 (0.60, 0.92)**	**0.001**	
Smoking						
No	1.00 (Ref)	**0.79 (0.65, 0.96)**	**0.61 (0.49, 0.76)**	**0.74 (0.60, 0.91)**	**< 0.001**	0.856
Yes	1.00 (Ref)	0.98 (0.58, 1.66)	0.65 (0.35, 1.19)	0.82 (0.46, 1.48)	0.297	
Wealth score index						
Low (< mean 70.45)	1.00 (Ref)	0.81 (0.64, 1.03)	**0.59 (0.45, 0.78)**	**0.70 (0.54, 0.91)**	**0.001**	**0.047**
High (> mean 70.45)	1.00 (Ref)	0.81 (0.61, 1.08)	**0.63 (0.47, 0.85)**	0.81 (0.60, 1.08)	0.060	
Physical activity						
Low (< mean 23.72)	1.00 (Ref)	**0.78 (0.62, 0.97)**	**0.62 (0.49, 0.80)**	0.82 (0.65, 1.03)	**0.019**	**< 0.001**
High (> mean 23.72)	1.00 (Ref)	0.94 (0.67, 1.32)	**0.61 (0.42, 0.87)**	**0.65 (0.45, 0.94)**	**0.003**	

*Note:* Significant *p*‐values are highlighted in bold. Multivariable logistic regression was used to estimate odds ratios (ORs) and 95% confidence intervals (CIs). Models were adjusted for age, sex, body mass index, physical activity, wealth score index, education level, smoking, hypertension, and dietary energy intake. For subgroup analyses, the stratifying variable was included as a main effect with a product (interaction) term, and the corresponding continuous covariate was omitted from the adjustment set for that subgroup to avoid over‐adjustment (e.g., age was not included when stratifying by age).

### 3.7 Sensitivity Analyses

Several sensitivity analyses were performed to evaluate the robustness of the findings (Table 6). The inverse association between PHDI adherence and hyperuricemia incidence persisted after additional adjustment for baseline HOMA‐IR, although the continuous estimate was marginally attenuated (OR per SD: 0.93; 95% CI: 0.87, 1.00; *p* = 0.060). The association remained statistically significant in analyses further adjusted for baseline C‐reactive protein (OR per SD: 0.91; 95% CI: 0.85, 0.98; *p* = 0.018), eGFR (OR per SD: 0.89; 95% Cl: 0.82, 0.95; *p* = 0.001), the triglyceride‐to‐HDL‐C ratio (OR per SD: 0.89; 95% CI: 0.83, 0.96; *p* = 0.003), baseline serum creatinine (OR per SD: 0.89; 95% CI: 0.82, 0.95; *p* = 0.001), and dietary protein intake expressed as a percentage of total energy (OR per SD: 0.91; 95% CI: 0.85, 0.98; *p* = 0.017). Excluding participants with baseline CKD (eGFR < 60 mL/min/1.73 m^2^) (*n* = 83 excluded) did not materially change the results (OR per SD: 0.89; 95% CI: 0.83, 0.96; Q4 vs. Q1 OR: 0.78; 95% CI: 0.64, 0.94; *p*‐trend < 0.001).

**TABLE 6:** Sensitivity analysis for the association between Planetary Health Diet Index and hyperuricemia incidence.

	Q1	Q2	Q3	Q4	*p*‐trend	Continues (Per standard deviation increment)	*p*
Excluding participants with CKD at baseline (*n* = 5690)^a^	1.00 (Ref)	0.84 (0.69, 1.01)	**0.64 (0.52, 0.78)**	**0.78 (0.64, 0.95)**	0.001	**0.89 (0.83, 0.96)**	0.003
Additional adjusted for baseline eGFR	1.00 (Ref)	**0.82 (0.68, 0.98)**	**0.62 (0.51, 0.76)**	**0.75 (0.62, 0.92)**	< 0.001	**0.89 (0.82, 0.95)**	0.001
Additional adjusted for baseline HOMA‐IR	1.00 (Ref)	0.88 (0.73, 1.06)	**0.75 (0.61, 0.92)**	0.84 (0.69, 1.02)	0.030	0.93 (0.87, 1.00)	0.060
Additional adjusted for baseline C‐reactive protein	1.00 (Ref)	0.89 (0.74, 1.06)	**0.71 (0.57, 0.87)**	**0.81 (0.66, 0.98)**	0.007	**0.91 (0.85, 0.98)**	0.018
Additional adjusted for baseline triglycerides/HDL‐C ratio	1.00 (Ref)	**0.83 (0.69, 0.99)**	**0.65 (0.53, 0.79)**	**0.77 (0.64, 0.94)**	0.001	**0.89 (0.83, 0.96)**	0.003
Additional adjusted for baseline creatinine	1.00 (Ref)	**0.82 (0.68, 0.98)**	**0.62 (0.51, 0.76)**	**0.76 (0.62, 0.92)**	< 0.001	**0.89 (0.82, 0.95)**	0.001
Additional adjusted for dietary protein intake (% of energy)	1.00 (Ref)	0.84 (0.70, 1.01)	**0.67 (0.55, 0.82)**	**0.81 (0.66, 0.98)**	0.006	**0.91 (0.85, 0.98)**	0.017

*Note:* Significant *p*‐values are highlighted in bold. Multivariable logistic regression was used to estimate odds ratios (ORs) and 95% confidence intervals (CIs). Models were adjusted age, sex, body mass index, physical activity, wealth score index, education level, smoking, hypertension, and dietary energy intake.

^a^ Chronic kidney disease (CKD) was defined as estimated glomerular filtration rate (eGFR) < 60 mL/min/1.73 m^2^ at baseline. This model excludes all participants with CKD at baseline (*n* = 83) and is adjusted for the same covariates as the primary Model 3, without further adjustment for eGFR.

Table S6 presents the association between PHDI adherence and incident hyperuricemia after additional adjustment for established dietary indices. The inverse association between the PHDI (per SD) and hyperuricemia persisted after adjustment for the Mediterranean Diet Score (OR: 0.91; 95% CI: 0.85–0.98; *p* = 0.017), the Healthy Eating Index 2015 (OR: 0.92; 95% CI: 0.85–0.99; *p* = 0.022), the Diet Quality Index Revised (OR: 0.91; 95% CI: 0.85–0.98; *p* = 0.014), and the DASH score (OR: 0.91; 95% CI: 0.85–0.98; *p* = 0.010). In categorical analyses, the highest versus lowest PHDI quartile remained significantly associated with lower hyperuricemia odds after adjustment for the Mediterranean Diet Score (Q4 OR: 0.80; 95% CI: 0.66–0.98; *p*‐trend = 0.005), HEI‐2015 (Q4 OR: 0.81; 95% CI: 0.67–0.99; *p*‐trend = 0.009), DQI‐R (Q4 OR: 0.80; 95% CI: 0.66–0.98; *p*‐trend = 0.006), and DASH score (Q4 OR: 0.79; 95% CI: 0.65–0.96; *p*‐trend = 0.003).

Adjustment for the E‐DII produced the greatest attenuation of the PHDI–hyperuricemia association. In the continuous analysis, the per‐SD OR was attenuated to 0.93 (95% CI: 0.86–1.00; *p* = 0.047), and in categorical analysis, the Q4 versus Q1 comparison became non‐significant (OR: 0.83; 95% CI: 0.68–1.01; *p* = 0.061), although the linear *p*‐trend across quartiles remained significant (*p*‐trend = 0.019).

### 3.8 Individual PHDI Components

Examination of individual PHDI components (Table S3) revealed that whole grains (OR per SD: 0.82; 95% CI: 0.76, 0.89), legumes (OR: 0.66; 95% CI: 0.60, 0.73), fruits (OR: 0.82; 95% CI: 0.76, 0.90), total vegetables (OR: 0.65; 95% CI: 0.58, 0.73), fish and seafood (OR: 0.87; 95% CI: 0.81, 0.94), dairy products (OR: 0.74; 95% CI: 0.67, 0.82), vegetable oils (OR: 0.86; 95% CI: 0.80, 0.93), and poultry (OR: 0.83; 95% CI: 0.76, 0.89) were each significantly associated with lower odds of hyperuricemia. Conversely, tubers and starchy vegetables (OR: 1.13; 95% CI: 1.06, 1.21) and added sugars (OR: 1.19; 95% CI: 1.10, 1.28) were positively associated with hyperuricemia risk.

### 3.9 Comparison With Alternative EAT‐Lancet Diet Indices

Application of alternative EAT‐Lancet diet indices corroborated the primary findings (Table S4). Using the binary Knuppel EAT‐Lancet Score, the highest versus lowest tertile was associated with 25% lower odds of hyperuricemia (OR: 0.75; 95% CI: 0.62, 0.91; *p*‐trend < 0.001). The Stubbendorff EAT‐Lancet Index, which employs a more granular scoring system, yielded a stronger inverse association, with 41% lower odds in the highest quartile (OR: 0.59; 95% CI: 0.46, 0.74; *p*‐trend < 0.001). In linear regression analyses (Table S5), each SD increment in the Knuppel EAT‐Lancet Score and the Stubbendorff EAT‐Lancet Index was associated with reductions in serum uric acid of 0.04 mg/dL (*p* = 0.003) and 0.08 mg/dL (*p* < 0.001), respectively, following full covariate adjustment.

## 4 Discussion

In this prospective analysis of 5773 Iranian adults followed for five years, we observed a consistent inverse association between adherence to the EAT‐Lancet planetary health diet and both serum uric acid levels and incident hyperuricemia. Moving from the lowest to the highest quartile of the PHDI was associated with a 25% reduction in hyperuricemia risk after full adjustment, and each standard deviation increment in the score corresponded to a measurable reduction in circulating uric acid.

To our knowledge, only one other study has examined the association between the PHDI and hyperuricemia. Tan et al., drawing on NHANES data from 22,087 American adults, reported that participants in the highest quartile of the PHDI for the United States (PHDI‐US) had 18% lower odds of hyperuricemia compared with those in the lowest quartile (OR 0.82, 95% CI 0.73–0.93) after multivariable adjustment, though this protective association was notably weaker than that observed for the Alternative Healthy Eating Index (Tan et al. 2025). Our study extends this finding to a non‐Western population with a fundamentally different dietary architecture—one where legumes and whole grains, rather than red meat, dominate the variation in PHDI scores. The fact that the inverse association with hyperuricemia held in an Iranian cohort with high refined grain consumption and relatively low dairy intake suggests the relationship is not confined to the American dietary context.

The dietary intake data presented in Table 2 illuminate the nutritional architecture underlying these associations. Across ascending PHDI quartiles, participants consumed progressively more dietary fiber (from 24.5 to 26.1 g/day), folate, vitamin C, magnesium, and potassium, while intakes of saturated fatty acids, trans fatty acids, and added sugars declined in parallel. These shifts reflect genuine displacement: as whole grains, legumes, fruits, and vegetables assumed a larger share of total energy, refined grains, red and processed meats, animal fats, and sugar‐sweetened products were proportionally reduced. This pattern of nutrient substitution is mechanistically relevant. Dietary fiber binds purines in the intestinal lumen, reducing their absorption and thereby limiting substrate availability for hepatic uric acid synthesis (Yu et al. 2025). The decline in added sugar intake—from 50.2 g/day in the lowest PHDI quartile to 38.2 g/day in the highest—carries particular significance, given that fructose metabolism in the liver catalyzes ATP depletion and nucleotide turnover, generating uric acid as a terminal byproduct through the activation of adenosine monophosphate deaminase and the purine degradation cascade (Zhang et al. 2022). Meanwhile, polyphenols and flavonoids concentrated in whole grains, legumes, fruits, and vegetables inhibit xanthine oxidoreductase, the rate‐limiting enzyme in uric acid production, through hydroxyl and planar structural motifs that directly bind the enzyme's active site (Li et al. 2024). The independent protective associations we observed for whole grains, legumes, fruits, total vegetables, fish, dairy products, and vegetable oils in component‐level analysis (Table S3) align with this mechanistic plurality. Dairy proteins—casein and lactalbumin—exert an independent uricosuric effect by increasing the fractional excretion of uric acid without altering glomerular filtration rate (Dalbeth et al. 2010), while the inverse associations for poultry and eggs likely reflect their displacement of red meat as the primary animal protein source in the upper PHDI quartiles. These component‐level results should, however, be interpreted cautiously because food groups are strongly correlated; a person who consumes more whole grains, for instance, tends to consume less refined grains and red meat, making it difficult to disentangle independent effects.

Critically, two components operated in the opposite direction. Tubers and starchy vegetables were positively associated with hyperuricemia risk (OR 1.13 per SD; 95% CI 1.06–1.21), likely reflecting the high glycemic response and insulinotropic effect of potatoes, which are the predominant tuber in the Iranian diet; insulin stimulates renal urate reabsorption via upregulation of URAT1 in the proximal tubule (Djousse et al. 2024). Added sugars showed the strongest detrimental association of any component (OR 1.19 per SD; 95% CI 1.10–1.28), consistent with the fructose‐driven ATP degradation pathway and the observation that sugar‐sweetened beverage consumption is among the most potent dietary risk factors for hyperuricemia identified in prospective cohorts (Lu et al. 2025).

We employed three EAT‐Lancet indices spanning distinct scoring philosophies to test whether the association with hyperuricemia depended on how adherence was operationalized. The Knuppel EAT‐Lancet Score uses a binary system—one point per met recommendation, 0 to 14 points (Knuppel et al. 2019)—while the Stubbendorff Index adopts a graded 0‐to‐3‐point scale per component (range 0–42) (Stubbendorff et al. 2022), and the PHDI employs fully proportional scoring across 16 components (0–150 points), including two ratio components—dark green vegetables and red‐orange vegetables, each expressed as a proportion of total vegetable intake—that are absent from the other indices (Cacau et al. 2021). The three did not perform identically. The Stubbendorff index showed the strongest association, with a 41% lower risk in the highest quartile (OR 0.59, 95% CI 0.46–0.74) and a 15% reduction per standard deviation (OR 0.85, 95% CI 0.79–0.92), followed by the PHDI (Q4 OR 0.75, 95% CI 0.62–0.91; per‐SD OR 0.88, 95% CI 0.82–0.95) and the Knuppel EAT‐Lancet Score, which yielded the weakest gradient (per‐SD OR 0.89, 95% CI 0.83–0.95) and a compressed observed range of just 1 to 8 points. This gradient in performance aligns with what one would expect from the underlying scoring structures. Binary cut‐offs, by design, treat all intake above a threshold as equivalent, discarding information about how far an individual exceeds or falls short of a target; the Stubbendorff index recovers some of this lost variation through its intermediate categories, while the PHDI preserves the full continuum. That the Stubbendorff index nonetheless outperformed the more detailed PHDI in our data suggests that scoring nuance, rather than the inclusion of additional components such as the vegetable ratio sub‐scores, was the primary driver of the stronger signal in this cohort. From a practical standpoint, graded scales like the Stubbendorff index may be better suited to detecting diet‐disease associations in populations where dietary contrasts are modest, whereas binary scores, however straightforward to implement in public health settings, risk underestimating the strength of the underlying relationship. The overall consistency in direction across all three indices, despite these differences in magnitude, reinforces the conclusion that the protective association with hyperuricemia is not an artifact of any single scoring method.

The inverse association between the PHDI and hyperuricemia was not uniform across all segments of the population, and several of the observed interactions warrant comment. The protective gradient was substantially stronger in women than in men (*p* for interaction < 0.001), with the highest PHDI quartile associated with a 32% reduction in odds among women compared with a non‐significant 17% among men. Endogenous estrogen promotes renal urate excretion by downregulating URAT1 and GLUT9 transporters in the proximal tubule, and this uricosuric effect may amplify the benefits of a diet already low in purine‐rich animal foods and fructose—essentially, the hormonal milieu and the dietary pattern pulling in the same direction (Halperin Kuhns and Woodward 2020). After menopause, when estrogen levels decline, this advantage would be expected to narrow, and our observation that the association attenuated in participants older than 45 years (*p* for interaction = 0.002) is consistent with that logic. Age‐related changes in glomerular filtration rate and a growing burden of subclinical kidney dysfunction in older adults may further blunt the capacity of dietary improvement to lower circulating urate (Wang et al. 2021). The weaker signal in participants with overweight and obesity (*p* for interaction < 0.001) points toward a third mechanism. Adipose tissue is a metabolically active source of xanthine oxidoreductase, the enzyme responsible for the terminal step in uric acid synthesis, and the low‐grade systemic inflammation that accompanies excess adiposity can impair renal urate clearance independently of diet (Furuhashi 2020). Under these conditions, even a well‐formulated diet may struggle to overcome the urate‐generating pressure of expanded fat mass. The absence of a clear dose–response gradient among smokers, while expected given that tobacco use independently elevates serum urate through nicotine‐induced oxidative stress and reduced renal blood flow (Li et al. 2025), should be interpreted cautiously because of the small proportion of smokers in this cohort. The stronger association among participants with lower socioeconomic status and higher physical activity suggests that the PHDI may confer particular benefit when it represents a genuine departure from the prevailing low‐quality dietary pattern—as is often the case in resource‐constrained settings—and that the urate‐lowering effects of diet and exercise may be additive. Whether these interactions replicate in cohorts with different distributions of these effect modifiers is a question worth pursuing, as the answer would sharpen our understanding of who stands to gain most from shifting toward a planetary health diet.

A persistent concern in observational studies linking diet to uric acid is that the association may be carried by underlying insulin resistance, subclinical inflammation, or impaired kidney function rather than by diet per se. We tested this directly by sequentially adjusting for markers of each of these pathways. Adding baseline HOMA‐IR to the fully adjusted model attenuated the per‐standard‐deviation estimate from 0.88 to 0.93 and rendered the trend across quartiles borderline (*p* = 0.030), which is precisely what one would expect if insulin resistance sits on the causal path between diet and urate. Our mediation analysis was consistent with this interpretation: the indirect pathway through HOMA‐IR accounted for a large portion of the association, whereas the direct effect, independent of this pathway, was virtually null. However, the wide 95% confidence interval for the proportion mediated (48.2% to 232.5%) urges caution; the point estimate of 91% may overstate the precision of this pathway, and the upper bound exceeding 100% hints at possible model instability or inconsistent mediation. Thus, while the findings support the notion that insulin sensitivity is a key intermediate, they cannot quantify its contribution with precision. The mechanism is biologically coherent. Insulin is a potent regulator of renal urate excretion; hyperinsulinemia stimulates URAT1 and other urate transporters in the proximal tubule, promoting the reabsorption of filtered urate back into the circulation (Toyoki et al. 2017). The attenuation observed upon adjusting for the dietary inflammatory index suggests that anti‐inflammatory pathways also play a supporting role, but the near‐complete mediation by HOMA‐IR points to insulin sensitivity as the central bridge connecting a sustainable dietary pattern to purine metabolism. This insight is clinically significant because it implies that the urate‐lowering benefits of the EAT‐Lancet diet will be most pronounced in individuals with some degree of insulin resistance—precisely the population at greatest need of dietary intervention.

The EAT‐Lancet diet is, after all, a dietary pattern that improves insulin sensitivity through its emphasis on whole grains, legumes, and fiber while restricting added sugars and refined carbohydrates (Lin et al. 2023); the sensitivity analyses indicating that the signal was more robust to adjustment for inflammation and kidney function than to HOMA‐IR is fully explained by this mediation structure. While the direct xanthine oxidase inhibition by dietary polyphenols and the uricosuric effect of dairy proteins and vitamin C are well‐documented, their contribution to the overall risk reduction in a free‐living population appears to run largely in parallel with, or be overshadowed by, the overriding influence of insulin sensitivity on renal urate handling.

Mutual adjustment analyses provided insight into whether the protective association of the PHDI with hyperuricemia represents a distinct dietary signal or largely reflects adherence to a generally healthy diet. When established dietary quality indices were added to the fully adjusted model, the PHDI–hyperuricemia association persisted, with the per‐SD OR attenuating modestly from 0.88 in the primary model to approximately 0.91–0.92 after adjustment for the Mediterranean Diet Score, HEI‐2015, DQI‐R, or DASH score, and quartile *p*‐trends remained significant in all cases. This indicates that while the PHDI shares variance with these conventional indices—unsurprisingly, given their overlapping emphasis on fruits, vegetables, whole grains, and legumes—it captures dietary dimensions relevant to uric acid homeostasis not fully represented by traditional healthy eating metrics. Conversely, adjustment for the E‐DII produced the greatest attenuation, with the per‐SD OR weakening to 0.93 (95% CI: 0.86–1.00; *p* = 0.047) and the Q4 versus Q1 comparison becoming non‐significant (*p* = 0.061), although the linear *p*‐trend remained significant (*p*‐trend = 0.019).

### 4.1 Strengths and Limitations

Several aspects of this study's design warrant consideration. A particular strength is our use of three distinct EAT‐Lancet indices—the PHDI, the EPIC‐Oxford EAT‐Lancet Score, and the Stubbendorff EAT‐Lancet Index—which span binary, graded, and continuous scoring systems. This was a deliberate choice. Comparative work has shown that these indices do not classify individuals identically, and concordance between them can be low (Miranda et al. 2025). That the inverse association with hyperuricemia held across all three formulations reduces the likelihood that our findings are an artifact of how the diet was operationalized. The dose–response gradient observed with the PHDI and the consistent direction of effect with the Knuppel and Stubbendorff indices further support a genuine relationship. Other strengths include the prospective design with five years of follow‐up, which strengthens the temporal clarity of the exposure‐outcome relationship. The incorporation of a formal, bootstrapped causal mediation analysis permitted us to move beyond describing the association and to quantify a specific biological pathway—insulin resistance—through which the PHDI exerts most of its protective effect. Hyperuricemia was defined by directly measured serum uric acid rather than self‐report, and incident cases required confirmation at a subsequent visit, reducing misclassification. The rich covariate set—including measures of adiposity, insulin resistance, kidney function, and inflammatory markers—allowed a careful sequence of adjustments and sensitivity analyses that address several alternative explanations.

Several limitations should be acknowledged. Firstly, reliance on a FFQ, however well validated, introduces measurement error that is inherent to all large‐scale nutritional epidemiology; FFQs systematically underestimate absolute intake of food groups relevant to the EAT‐Lancet framework—particularly nuts, seeds, and vegetable oils—which would attenuate associations but also means our absolute PHDI scores are not directly comparable to those from 24‐h recall‐based studies. Secondly, residual confounding cannot be ruled out, as individuals who score highly on dietary indices tend to differ from those who do not in ways that extend beyond measured covariates, and while our sensitivity analyses adjusting for HOMA‐IR, C‐reactive protein, and the triglyceride‐to‐HDL ratio did not materially alter the results, some of the protective association may still reflect broader health‐seeking behavior. Thirdly, the study population—an Iranian cohort with high refined grain and tea consumption and comparatively low red meat and dairy intake relative to Western populations—is simultaneously a strength and a limitation: it allowed us to test the EAT‐Lancet framework in a setting where legumes and whole grains rather than red meat drove score variation, but findings may not generalize to populations with fundamentally different dietary structures. Finally, the observational design precludes causal claims, although the exclusion of prevalent hyperuricemia at baseline and the consistency of findings across strata of kidney function argue against a strong reverse causation story.

## 5 Conclusion

In this prospective cohort of Iranian adults, greater adherence to the EAT–Lancet planetary health diet was associated with lower follow‐up serum uric acid levels and reduced odds of incident hyperuricemia over five years. The data are consistent with a pathway in which the diet's influence on insulin sensitivity plays a central role in this relationship, though the precise extent of mediation could not be determined with certainty. The protective signal, consistent across three distinct scoring systems, appears to arise not merely from the avoidance of purine‐rich foods but from a broader shift in metabolic physiology—a recalibration of the insulin–urate axis in the kidney. Our findings suggest that the planetary health diet's capacity to improve insulin sensitivity may be an important lever through which it is linked to lower serum uric acid, positioning this dietary framework not just as a strategy for environmental stewardship and chronic disease prevention, but as a potentially relevant nutritional approach for addressing the metabolic derangements that drive hyperuricemia. Whether this pathway translates into fewer gout attacks or slower progression of urate‐associated nephropathy in clinical populations remains a key question for future interventional and longer‐term prospective studies.

## Author Contributions

**Farzam Kamrani:** formal analysis, writing – original draft, conceptualization. **Mobina Imannezhad:** writing – original draft. **Elahe Etesami:** writing – original draft. **Elham karimi:** writing – original draft. **Matin Ghanavati:** writing – review and editing. **Jalaledin Mirzay Razaz:** supervision. **Reza Homayounfar:** supervision, writing – review and editing. **Ali Nikparast:** formal analysis, conceptualization.

## Funding

The authors have nothing to report.

## Ethics Statement

The National Nutrition and Food Technology Research Institute (NNFTRI) ethics committee approved the study protocol (Ethics code: IR.SBMU.NNFTRI.REC.1404.003). All participants provided written informed consent and were informed about the study. All procedures performed in studies involving human participants adhered to the ethical standards of the institutional and/or national research committee and to the 1964 Helsinki Declaration and its later amendments, or to comparable ethical standards.

## Consent

The authors have nothing to report.

## Conflicts of Interest

The authors declare no conflicts of interest.

## Supporting information


**Table S1:** Composition of Planetary Health Diet components based on FFQ items in the present study.
**Table S2:** Planetary Health Diet scoring criteria and cut‐offs.
**Table S3:** Individual Planetary Health Diet Index components and hyperuricemia incidence.
**Table S4:** Odds ratios (95% confidence intervals) for hyperuricemia incidence according to adherence to the other EAT‐lancet diet indices.
**Table S5:** Unstandardized coefficients for serum uric acid levels according to each SD increase of the EAT‐lancet diet indices.
**Table S6:** Association between PHDI and incident hyperuricemia after additional adjustment for alternative dietary indices.
**Table S7:** Calculation methods for comparator dietary indices.

## Data Availability

The datasets analyzed in the current study are available from the corresponding author on reasonable request.

## References

[fsn372279-bib-0001] Amini, M. , M. Moradinazar , F. Rajati , et al. 2022. “Socioeconomic Inequalities in Prevalence, Awareness, Treatment and Control of Hypertension: Evidence From the PERSIAN Cohort Study.” BMC Public Health 22, no. 1: 1401. 35864469 10.1186/s12889-022-13444-x PMC9306154

[fsn372279-bib-0002] Cacau, L. T. , E. De Carli , A. M. de Carvalho , et al. 2021. “Development and Validation of an Index Based on EAT‐Lancet Recommendations: The Planetary Health Diet Index.” Nutrients 13, no. 5: 1698. 34067774 10.3390/nu13051698 PMC8156093

[fsn372279-bib-0003] Can, L. , L. Xiaolong , and L. Feifei . 2023. “Dietary Patterns and Hyperuricemia in Adult Subjects: A Systematic Review and Meta‐Analysis of Observational Studies.” World Journal of Public Health 8, no. 2: 55–67.

[fsn372279-bib-0004] Cheng, S. , L. Shan , Z. You , et al. 2023. “Dietary Patterns, Uric Acid Levels, and Hyperuricemia: A Systematic Review and Meta‐Analysis.” Food & Function 14, no. 17: 7853–7868. 37599588 10.1039/d3fo02004e

[fsn372279-bib-0005] Dalbeth, N. , S. Wong , G. D. Gamble , et al. 2010. “Acute Effect of Milk on Serum Urate Concentrations: A Randomised Controlled Crossover Trial.” Annals of the Rheumatic Diseases 69, no. 9: 1677–1682. 20472590 10.1136/ard.2009.124230

[fsn372279-bib-0006] Djousse, L. , X. Zhou , J. Lim , et al. 2024. “Potato Consumption and Risk of Type 2 Diabetes Mellitus: A Harmonized Analysis of 7 Prospective Cohorts.” Journal of Nutrition 154, no. 10: 3079–3087. 39289134 10.1016/j.tjnut.2024.07.020 PMC12612587

[fsn372279-bib-0007] Du, L. , Y. Zong , H. Li , et al. 2024. “Hyperuricemia and Its Related Diseases: Mechanisms and Advances in Therapy.” Signal Transduction and Targeted Therapy 9, no. 1: 212. 39191722 10.1038/s41392-024-01916-y PMC11350024

[fsn372279-bib-0008] Eghtesad, S. , A. Hekmatdoost , E. Faramarzi , et al. 2023. “Validity and Reproducibility of a Food Frequency Questionnaire Assessing Food Group Intake in the PERSIAN Cohort Study.” Frontiers in Nutrition 10: 1059870. 37599697 10.3389/fnut.2023.1059870 PMC10436288

[fsn372279-bib-0009] Emami, O. , A. Nikparast , M. Sepehrinia , S. Hadi , J. Mirzay Razzaz , and R. Homayounfar . 2025. “Ultra‐Processed Food Consumption and the Risk of Developing Metabolic Dysfunction‐Associated Steatotic Liver Disease (MASLD): A Five‐Year Prospective Cohort Study in Iranian Adults.” Journal of Health, Population and Nutrition 44, no. 1: 409. 41291958 10.1186/s41043-025-01150-4 PMC12648998

[fsn372279-bib-0010] Fung, T. T. , S. E. Chiuve , M. L. McCullough , K. M. Rexrode , G. Logroscino , and F. B. Hu . 2008. “Adherence to a DASH‐Style Diet and Risk of Coronary Heart Disease and Stroke in Women.” Archives of Internal Medicine 168, no. 7: 713–720. 18413553 10.1001/archinte.168.7.713

[fsn372279-bib-0011] Furuhashi, M. 2020. “New Insights Into Purine Metabolism in Metabolic Diseases: Role of Xanthine Oxidoreductase Activity.” American Journal of Physiology, Endocrinology and Metabolism 319, no. 5: E827–E834. 32893671 10.1152/ajpendo.00378.2020

[fsn372279-bib-0012] Giles, T. D. , B. J. Materson , J. N. Cohn , and J. B. Kostis . 2009. “Definition and Classification of Hypertension: An Update.” Journal of Clinical Hypertension 11, no. 11: 611–614. 19878368 10.1111/j.1751-7176.2009.00179.x PMC8673286

[fsn372279-bib-0013] Haines, P. S. , A. M. Siega‐Riz , and B. M. Popkin . 1999. “The Diet Quality Index Revised: A Measurement Instrument for Populations.” Journal of the American Dietetic Association 99, no. 6: 697–704. 10361532 10.1016/S0002-8223(99)00168-6

[fsn372279-bib-0014] Halperin Kuhns, V. L. , and O. M. Woodward . 2020. “Sex Differences in Urate Handling.” International Journal of Molecular Sciences 21, no. 12: 4269. 32560040 10.3390/ijms21124269 PMC7349092

[fsn372279-bib-0015] Johnson, R. J. , L. G. S. Lozada , M. A. Lanaspa , F. Piani , and C. Borghi . 2023. “Uric Acid and Chronic Kidney Disease: Still More to Do.” Kidney International Reports 8, no. 2: 229–239. 36815099 10.1016/j.ekir.2022.11.016 PMC9939362

[fsn372279-bib-0016] Knuppel, A. , K. Papier , T. J. Key , and R. C. Travis . 2019. “EAT‐Lancet Score and Major Health Outcomes: The EPIC‐Oxford Study.” Lancet 394, no. 10194: 213–214. 31235280 10.1016/S0140-6736(19)31236-X

[fsn372279-bib-0017] Krebs‐Smith, S. M. , T. E. Pannucci , A. F. Subar , et al. 2018. “Update of the Healthy Eating Index: HEI‐2015.” Journal of the Academy of Nutrition and Dietetics 118, no. 9: 1591–1602. 30146071 10.1016/j.jand.2018.05.021 PMC6719291

[fsn372279-bib-0018] Li, K. , Y. Wang , W. Liu , et al. 2024. “Structure‐Activity Relationships and Changes in the Inhibition of Xanthine Oxidase by Polyphenols: A Review.” Food 13, no. 15: 2365. 10.3390/foods13152365 PMC11312107 39123556

[fsn372279-bib-0019] Li, P. , X. Li , G. Li , et al. 2025. “The Association Between Smoking and the Occurrence of Hyperuricemia: A Retrospective Cohort Study.” Tobacco Induced Diseases 23. 10.18332/tid/204253. PMC12124120 40453634

[fsn372279-bib-0020] Lin, X. , S. Wang , and J. Huang . 2023. “The Association Between the EAT–Lancet Diet and Diabetes: A Systematic Review.” Nutrients 15, no. 20: 4462. 37892537 10.3390/nu15204462 PMC10610026

[fsn372279-bib-0021] Lu, Y. , Y. Wang , R. Huang , et al. 2025. “Sugar‐Sweetened Beverages and the Risk of Hyperuricemia and Gout: A Meta‐Analysis.” Frontiers in Nutrition 12: 1669129. 41190154 10.3389/fnut.2025.1669129 PMC12580599

[fsn372279-bib-0022] Ming, Y. , Z. Sun , M. Tao , et al. 2026. “Association Between the Planetary Health Diet Index and Chronic Kidney Disease Prevalence and Mortality: An Analysis of NHANES 2005–2018.” Food Science & Nutrition 14, no. 1: e71402. 41458533 10.1002/fsn3.71402 PMC12743143

[fsn372279-bib-0023] Miranda, A. R. , F. Vieux , M. Maillot , and E. O. Verger . 2025. “How Do the Indices Based on the EAT‐Lancet Recommendations Measure Adherence to Healthy and Sustainable Diets? A Comparison of Measurement Performance in Adults From a French National Survey.” Current Developments in Nutrition 9, no. 3: 104565. 40104607 10.1016/j.cdnut.2025.104565 PMC11919322

[fsn372279-bib-0024] Modification of Diet in Renal Disease Study Group . 1992. “The Modification of Diet in Renal Disease Study: Design, Methods, and Results From the Feasibility Study.” American Journal of Kidney Diseases 20, no. 1: 18–33. 1621675 10.1016/s0272-6386(12)80313-1

[fsn372279-bib-0025] Momenan, A. A. , M. Delshad , N. Sarbazi , G. N. Rezaei , A. Ghanbarian , and F. Azizi . 2012. “Reliability and Validity of the Modifiable Activity Questionnaire (MAQ) in an Iranian Urban Adult Population.” Arch Iran Med 15, no. 5: 279–282. 22519376

[fsn372279-bib-0026] Ngandeu‐Singwe, M. , J. R. Nkeck , A. Hamroun , et al. 2026. “Worldwide Trends in Hyperuricaemia From 2000 to 2023: A Systematic Review and Modelling Analysis.” Lancet Rheumatology 8, no. 5: e346–e362. 41662849 10.1016/S2665-9913(25)00344-3

[fsn372279-bib-0027] Ruiz‐García, A. , A. Serrano‐Cumplido , E. Arranz‐Martínez , C. Escobar‐Cervantes , and V. Pallarés‐Carratalá . 2024. “Hyperuricaemia Prevalence Rates According to Their Physiochemical and Epidemiological Diagnostic Criteria and Their Associations With Cardio‐Renal‐Metabolic Factors: SIMETAP‐HU Study.” Journal of Clinical Medicine 13, no. 16: 4884. 39201026 10.3390/jcm13164884 PMC11355702

[fsn372279-bib-0028] Shivappa, N. , S. E. Steck , T. G. Hurley , J. R. Hussey , and J. R. Hébert . 2014. “Designing and Developing a Literature‐Derived, Population‐Based Dietary Inflammatory Index.” Public Health Nutrition 17, no. 8: 1689–1696. 23941862 10.1017/S1368980013002115 PMC3925198

[fsn372279-bib-0029] Stubbendorff, A. , E. Sonestedt , S. Ramne , I. Drake , E. Hallström , and U. Ericson . 2022. “Development of an EAT‐Lancet Index and Its Relation to Mortality in a Swedish Population.” American Journal of Clinical Nutrition 115, no. 3: 705–716. 34791011 10.1093/ajcn/nqab369 PMC8895215

[fsn372279-bib-0030] Tan, J.‐X. , Z. Qin , Q.‐Z. Li , G.‐T. Ruan , and Y.‐Z. Gong . 2025. “Association and Optimization of the Planetary Health Diet Index With Hyperuricemia.” Frontiers in Nutrition 12: 1657218. 41031353 10.3389/fnut.2025.1657218 PMC12477380

[fsn372279-bib-0031] Toyoki, D. , S. Shibata , E. Kuribayashi‐Okuma , et al. 2017. “Insulin Stimulates Uric Acid Reabsorption via Regulating Urate Transporter 1 and ATP‐Binding Cassette Subfamily G Member 2.” American Journal of Physiology‐Renal Physiology 313, no. 3: F826–F834. 28679589 10.1152/ajprenal.00012.2017

[fsn372279-bib-0032] Trichopoulou, A. , T. Costacou , C. Bamia , and D. Trichopoulos . 2003. “Adherence to a Mediterranean Diet and Survival in a Greek Population.” New England Journal of Medicine 348, no. 26: 2599–2608. 12826634 10.1056/NEJMoa025039

[fsn372279-bib-0033] VanItallie, T. , M.‐U. Yang , S. B. Heymsfield , R. C. Funk , and R. A. Boileau . 1990. “Height‐Normalized Indices of the Body's Fat‐Free Mass and Fat Mass: Potentially Useful Indicators of Nutritional Status.” American Journal of Clinical Nutrition 52, no. 6: 953–959. 2239792 10.1093/ajcn/52.6.953

[fsn372279-bib-0034] Wang, Y. , W. Zhang , T. Qian , et al. 2021. “Reduced Renal Function May Explain the Higher Prevalence of Hyperuricemia in Older People.” Scientific Reports 11, no. 1: 1302. 33446773 10.1038/s41598-020-80250-z PMC7809022

[fsn372279-bib-0035] Wen, Z.‐Y. , Y.‐F. Wei , Y.‐H. Sun , and W.‐P. Ji . 2024. “Dietary Pattern and Risk of Hyperuricemia: An Updated Systematic Review and Meta‐Analysis of Observational Studies.” Frontiers in Nutrition 11: 1218912. 38481974 10.3389/fnut.2024.1218912 PMC10932991

[fsn372279-bib-0036] Willett, W. , J. Rockström , B. Loken , et al. 2019. “Food in the Anthropocene: The EAT–Lancet Commission on Healthy Diets From Sustainable Food Systems.” Lancet 393, no. 10170: 447–492. 30660336 10.1016/S0140-6736(18)31788-4

[fsn372279-bib-0037] Willett, W. C. , L. Sampson , M. J. Stampfer , et al. 1985. “Reproducibility and Validity of a Semiquantitative Food Frequency Questionnaire.” American Journal of Epidemiology 122, no. 1: 51–65. 4014201 10.1093/oxfordjournals.aje.a114086

[fsn372279-bib-0038] Yang, S. , Y. Huang , Z. Ye , et al. 2026. “The EAT–Lancet Planetary Health Diet and Risk of Incident Chronic Kidney Disease.” CMAJ 198, no. 3: E73–E83. 41587806 10.1503/cmaj.250457 PMC12858240

[fsn372279-bib-0039] Yu, W. , J. Liu , D. Baranenko , et al. 2025. “The Role of Dietary Polysaccharides in Uric Acid Regulation: Mechanisms and Benefits in Managing Hyperuricemia.” Trends in Food Science & Technology 157: 104902.

[fsn372279-bib-0040] Zhang, P. , H. Sun , X. Cheng , et al. 2022. “Dietary Intake of Fructose Increases Purine De Novo Synthesis: A Crucial Mechanism for Hyperuricemia.” Frontiers in Nutrition 9: 1045805. 36601078 10.3389/fnut.2022.1045805 PMC9807165

[fsn372279-bib-0041] Zhang, Z. , S. Han , X. Ye , et al. 2026. “Adherence to the EAT‐Lancet Diet and Risk of Chronic Kidney Disease Among Middle‐Aged and Older Adults: Insights From Two Nationwide Cohort Studies.” Nutrition Journal 25, no. 1: 38. 41761272 10.1186/s12937-025-01236-z PMC13049795

