# Supplementary material for: Association Between the Planetary Health Diet Index and Serum Uric Acid Levels and Incident Hyperuricemia: Findings From an Iranian Prospective Cohort Study
Source: Food Sci Nutr. 2026 Aug 23;14(8):e72279. doi: 10.1002/fsn3.72279 (PMC13501001; doi:10.1002/fsn3.72279)
Supplement: Supplementary file 1 [file FSN3-14-e72279-s001.docx]

**Supplementary Table Legend:**

**Supplementary Table S1.** Composition of Planetary Health Diet components based on FFQ items in the present study

**Supplementary Table S2.** Planetary Health Diet scoring criteria and cut-offs

**Supplementary Table S3.** Individual Planetary Health Diet Index components and hyperuricemia incidence.

**Supplementary Table S4.** Odds ratios (95% confidence intervals) for hyperuricemia incidence according to adherence to the other EAT-Lancet Diet indices.

**Supplementary Table S5.** Unstandardized coefficients for serum uric acid levels according to each SD increase of the EAT-Lancet Diet Indices.

**Supplementary Table S6.** Association between PHDI and incident hyperuricemia after additional adjustment for alternative dietary indices.

**Supplementary Table S7.** Calculation Methods for Comparator Dietary Indices

**Supplementary Table S1.** Composition of Planetary Health Diet components based on FFQ items in the present study

| **PHDI Component** | **Scoring Category** | **FFQ Food Items Included** |
| --- | --- | --- |
| **Whole grains** | Adequacy | Bread (Barbari, Sangak), wheat, oats, barley |
| **Legumes** | Adequacy | Beans, chickpeas, mung beans, lentils, dhal, split peas, soybean, cooked fava beans, lima beans |
| **Nuts and seeds** | Adequacy | Walnuts, peanuts, almonds, pistachios, hazelnuts, pumpkin seeds, watermelon seeds, sunflower seeds |
| **Fruits (fresh and dried)** | Adequacy | Cantaloupe, honeydew, watermelon, apricot, cherries, nectarines, peaches, prunes, berries, strawberries, plums, figs, grapes, pears, apples, kiwifruit, citrus fruits, pomegranate, banana, persimmon, dates, fresh fruit juice, dried fruit, raisins |
| **Total vegetables** | Adequacy | Lettuce, cabbage, cauliflower, broccoli, tomatoes, cucumbers, leafy greens (fresh and cooked), eggplant, celery, beets, turnips, potatoes†, carrots, garlic, onions, bell peppers, mushrooms, corn, green peas, green beans, zucchini |
| **Dark green vegetables (DGV)** | Ratio | Lettuce, fresh leafy greens, cooked leafy greens, cabbage, cauliflower, broccoli |
| **Red and orange vegetables (ReV)** | Ratio | Tomatoes, carrots, bell peppers |
| **Tubers and starchy vegetables** | Optimum | Potatoes† |
| **Fish and seafood** | Optimum | Fish, canned tuna |
| **Eggs** | Optimum | Eggs |
| **Dairy products** | Optimum | Low-fat milk, high-fat milk, low-fat yogurt, high-fat yogurt, cheese, creamy cheese (Khameie), doogh/ayran, kashk (whey) |
| **Vegetable oils** | Optimum | Liquid oils, olive oil |
| **Red meat** | Moderation | Red meat (any kind), hamburger |
| **Poultry** | Moderation | Chicken, chicken giblets |
| **Processed meat and organ meats‡** | Included within red meat component | Sausages, salami, variety meats (heart, liver, kidney), sheep brain, tongue, tripe, leg/trotter |
| **Animal fats** | Moderation | Butter, hydrogenated animal fats |
| **Added sugars** | Moderation | Sugar cubes, sweetened beverages, jams, honey, pastries, and sweets. |

**Footnotes**

*Refined grains were included in total energy intake but were not scored as independent PHDI components.*

†Potatoes were treated as a separate “tubers” component in accordance with the PHDI scoring system. *

‡Processed meats and organ meats were categorized under the red meat component, consistent with the PHDI framework. *

**Supplementary Table S2.** Planetary Health Diet scoring criteria and cut-offs

All values represent the caloric contribution of each food group as a percentage of total energy intake (% of energy).

| **Component Category** | **Component** | **0 Points** | **5 Points** | **10 Points** | **5 Points** | **0 Points** |
| --- | --- | --- | --- | --- | --- | --- |
| **Adequacy components** | Nuts and peanuts | 0.0 | 🡨----🡪 | ≥11.6 | – | – |
|  | Legumes† | 0.0 | 🡨----🡪 | ≥11.3 | – | – |
|  | Fruits | 0.0 | 🡨----🡪 | ≥5.0 | – | – |
|  | Vegetables | 0.0 | 🡨----🡪 | ≥3.1 | – | – |
|  | Whole cereals | 0.0 | 🡨----🡪 | ≥32.4 | – | – |
| **Optimum components** | Eggs | 0.0 | 🡨----🡪 | 0.8 | 🡨----🡪 | ≥1.5 |
|  | Fish and seafood | 0.0 | 🡨----🡪 | 1.6 | 🡨----🡪 | ≥5.7 |
|  | Tubers and potatoes | 0.0 | 🡨----🡪 | 1.6 | 🡨----🡪 | ≥3.1 |
|  | Dairy§ | 0.0 | 🡨----🡪 | 6.1 | 🡨----🡪 | ≥12.2 |
|  | Vegetable oils° | 0.0 | 🡨----🡪 | 16.5 | 🡨----🡪 | ≥30.7 |
| **Ratio components** | DGV/total ratio‡ | 0.0 | 🡨--🡪29.5 | 29.5 | 🡨----🡪 | 100 |
|  | REV/total ratio≡ | 0.0 | 🡨--🡪38.5 | 38.5 | 🡨----🡪 | 100 |
| **Moderation components** | Red meat£ | ≥2.4 | 🡨----🡪 | 0.0 | – | – |
|  | Chicken and substitutes | ≥5.0 | 🡨----🡪 | 0.0 | – | – |
|  | Animal fats¥ | ≥1.4 | 🡨----🡪 | 0.0 | – | – |
|  | Added sugars | ≥4.8 | 🡨----🡪 | 0.0 | – | – |

**Scoring Notes**

- Adequacy components: Scored proportionally from 0 to 10 points, with higher intake receiving higher scores.
- Optimum components: Scored from 0 to 10 points, with maximum score assigned within the optimal intake range and decreasing toward both extremes.
- Ratio components:
  - DGV/total ratio = (dark green vegetables ÷ total vegetables) × 100
  - REV/total ratio = (red and orange vegetables ÷ total vegetables) × 100
- Moderation components: Reverse scored; higher intake receives lower scores (0 points at or above the specified threshold).

Abbreviations

- DGV: Dark Green Vegetables
- REV: Red and Orange Vegetables
- † Legumes: beans and soy (excluding dairy fats)
- § Dairy: excluding dairy fats
- ° Unsaturated oils
- £ Red meat: beef, lamb, and pork
- ¥ Animal fat: lard, tallow, and dairy fats

**Supplementary Table S3.** Individual Planetary Health Diet Index components and hyperuricemia incidence.

| **PHDI Component (Per SD increment)** | **OR (95% CI)** | **P-value** |
| --- | --- | --- |
| Whole grains | **0.82 (0.76, 0.89)** | <0.001 |
| Legumes | **0.66 (0.60, 0.73)** | <0.001 |
| Nuts and seeds | 0.97 (0.91, 1.05) | 0.474 |
| Fruits (fresh and dried) | **0.82 (0.76, 0.90)** | <0.001 |
| Total vegetables | **0.65 (0.58, 0.73)** | <0.001 |
| Dark green vegetables (DGV)/ total ratio | 0.96 (0.90, 1.03) | 0.284 |
| Red and orange vegetables (ReV)/ total ratio | **0.90 (0.84, 0.97)** | 0.004 |
| Tubers and starchy vegetables | **1.13 (1.06, 1.21)** | 0.001 |
| Fish and seafood | **0.87 (0.81, 0.94)** | 0.001 |
| Eggs | **0.92 (0.85, 0.99)** | 0.031 |
| Dairy products | **0.74 (0.67, 0.82)** | <0.001 |
| Vegetable oils | **0.86 (0.80, 0.93)** | <0.001 |
| Red meat† | 1.06 (0.98, 1.14) | 0.148 |
| Poultry | **0.83 (0.76, 0.89)** | <0.001 |
| Animal fats | 1.04 (0.96, 1.12) | 0.338 |
| Added sugars | **1.19 (1.10, 1.28)** | <0.001 |

**Footnotes:**
Model adjusted for age, sex, body mass index, physical activity, wealth score index, education level, smoking, hypertension, and dietary energy intake.

**Supplementary Table S4.** Odds ratios (95% confidence intervals) for hyperuricemia incidence according to adherence to the other EAT-Lancet Diet indices.

|  | Q1/ T1 | Q2/ T2 | Q3/ T3 | Q4 | P-trend | Continues  (Per SD increment) | P-value |
| --- | --- | --- | --- | --- | --- | --- | --- |
| **Knuppel EAT-Lancet Score (EPIC-Oxford study)** | | | | | | | |
| Median (Min–Max) | 3 (1–3) | 4 (4) | 5 (5–8) | — |  |  |  |
| Cases/populations | 482/1936 | 419/2543 | 212/1294 | — |  |  |  |
| Model 3 | 1.00 (Ref) | **0.74 (0.64, 0.87)** | **0.75 (0.62, 0.91)** | — | <0.001 | **0.89 (0.83, 0.95)** | 0.001 |
| **Stubbendorff EAT-Lancet Index** | | | | | | | |
| Median (IQR) | 17 (2) | 19 (0) | 20 (1) | 23 (2) |  |  |  |
| Cases/populations | 511/2072 | 169/995 | 308/1668 | 125/1038 |  |  |  |
| Model 3 | 1.00 (Ref) | **0.70 (0.57, 0.85)** | **0.81 (0.68, 0.97)** | **0.59 (0.46, 0.74)** | <0.001 | **0.85 (0.79, 0.92)** | <0.001 |
| Model 3 adjusted for age, sex, body mass index, physical activity, wealth score index, education level, smoking, hypertension, and dietary energy intake (kcal/day).  For the EAT-Lancet Score (EPIC-Oxford), the distribution permitted only 3 categories (tertiles) to be generated. The binary scoring method yields integer values on a theoretical 0–14 scale; however, in this study population, observed scores ranged from 1 to 8. Due to ties at specific integer values, the data lacked sufficient granularity to form 4 distinct quartile groups. Accordingly, categories are presented as tertiles (T1–T3). Furthermore, the interquartile range was zero for all tertile groups owing to the discrete integer scale; therefore, the distribution is presented as Median (Min–Max). | | | | | | | |

|  | **B** | **P-value** |
| --- | --- | --- |
| **EAT-Lancet Score (EPIC-Oxford study)** |  |  |
| Model 3 | **−0.044 (-0.073, -0.014** | 0.003 |
| **Stubbendorff EAT-Lancet Index** |  |  |
| Model 3 | **-0.085 (-0.116,**  **-0.054)** | <0.001 |

**Supplementary Table S5.** Unstandardized coefficients for serum uric acid levels according to each SD increase of the EAT-Lancet Diet Indices.

**Footnotes**

Model 3 adjusted for the baseline serum uric acid concentration, age, sex, body mass index, physical activity, wealth score index, education level, smoking, hypertension, and dietary energy intake (kcal/day). The unstandardized coefficient (B) represents the change in serum uric acid (mg/dL) per one standard deviation increment in the respective EAT-Lancet Diet Index score.

**Supplementary Table S6.** Association between PHDI and incident hyperuricemia after additional adjustment for alternative dietary indices**.**

| Dietary Index Added to Model 3 | Q1 (Ref) | Q2 OR (95% CI) | Q3 OR (95% CI) | Q4 OR (95% CI) | P-trend | Per SD OR (95% CI) | P-value |
| --- | --- | --- | --- | --- | --- | --- | --- |
| None (Model 3, primary) | 1.00 | **0.81 (0.68, 0.98)** | **0.62 (0.51, 0.76)** | **0.75 (0.62, 0.91)** | <0.001 | **0.88 (0.82, 0.95)** | 0.001 |
| + Mediterranean diet score | 1.00 | 0.87 (0.72, 1.04) | **0.69 (0.56, 0.85)** | **0.80 (0.66, 0.98)** | 0.005 | **0.91 (0.85, 0.98)** | 0.017 |
| + Healthy Eating Index 2015 | 1.00 | 0.89 (0.74, 1.07) | **0.71 (0.57, 0.87)** | **0.81 (0.67, 0.99)** | 0.009 | **0.92 (0.85, 0.99)** | 0.022 |
| + Diet Quality Index Revised | 1.00 | 0.86 (0.72, 1.04) | **0.70 (0.57, 0.86)** | **0.80 (0.66, 0.98)** | 0.006 | **0.91 (0.85, 0.98)** | 0.014 |
| + EDII | 1.00 | 0.88 (0.73, 1.06) | **0.73 (0.59, 0.89)** | 0.83 (0.68, 1.01) | 0.019 | **0.93 (0.86, 1.00)** | 0.047 |
| + DASH Diet Score | 1.00 | 0.86 (0.71, 1.03) | **0.68 (0.55, 0.83)** | **0.79 (0.65, 0.96)** | 0.003 | **0.91 (0.85, 0.98)** | 0.010 |

**Footnotes**

Model 3 is adjusted for age, sex, BMI, physical activity, Wealth Score Index, educational level, smoking status, hypertension, and total energy intake. Each row represents a separate model with the named dietary index added as an additional continuous covariate (per standard deviation). P-trend values are from linear modeling of PHDI quartiles. OR, odds ratio; CI, confidence interval; SD, standard deviation; HEI-2015, Healthy Eating Index 2015; DQI-R, Diet Quality Index Revised; EDII, Empirical Dietary Index for Hyperinsulinemia; DASH, Dietary Approaches to Stop Hypertension. Full scoring protocols for comparator dietary indices are provided in Supplementary Methods S7.

## Supplementary Table S7. Calculation Methods for Comparator Dietary Indices

| Index | Components (n) | Food Groups / Nutrients Included | Scoring System | Score Range | Calculation Method | Key Adaptation for Iranian FFQ | Reference |
| --- | --- | --- | --- | --- | --- | --- | --- |
| **Mediterranean Diet Score** | 9 | Vegetables; legumes; fruits & nuts; cereals; fish; dairy; meat & poultry; alcohol; MUFA:SFA ratio | Binary (sex-specific median cutoffs). Beneficial components (vegetables, legumes, fruits & nuts, cereals, fish): 1 point if intake ≥ median. Detrimental components (meat, dairy): 1 point if intake < median. MUFA: SFA ratio: 1 point if ≥ median. Alcohol: 1 point if intake between 10-50 g/d for men, 5-25 g/d for women. | 0–9 | Sum of 9 binary components. Higher scores indicate greater adherence to traditional Mediterranean diet. | Alcohol component excluded (prevalence near zero in Iranian cohort). MUFA: SFA ratio calculated from FFQ fatty acid data using USDA and Iranian Food Composition Tables. 8-component modified version used; range 0–8. | Trichopoulou et al., 2003 |
| **Healthy Eating Index 2015 (HEI-2015)** | 13 | **Adequacy:** Total fruits (0.8 cup eq/1000 kcal); whole fruits (0.4 cup eq/1000 kcal); total vegetables (1.1 cup eq/1000 kcal); greens & beans (0.2 cup eq/1000 kcal); whole grains (1.5 oz eq/1000 kcal); dairy (1.3 cup eq/1000 kcal); total protein foods (2.5 oz eq/1000 kcal); seafood & plant proteins (0.8 oz eq/1000 kcal); fatty acid ratio [(PUFA+MUFA)/SFA ≥ 2.5]; **Moderation:** refined grains (≤1.8 oz eq/1000 kcal); sodium (≤1.1 g/1000 kcal); added sugars (≤6.5% energy); saturated fats (≤8% energy) | Density-based (per 1,000 kcal or % energy). Each component scored proportionally between 0 (minimum standard) and maximum points (5 or 10) based on predefined thresholds. Scores between min and max are prorated. | 0–100 | Sum of 13 component scores. Higher scores indicate better alignment with the 2015-2020 Dietary Guidelines for Americans. | Sodium intake estimated from FFQ without discretionary salt; may be underestimated. Fatty acid ratio calculated as (MUFA + PUFA) / SFA. Added sugars estimated from sugar-sweetened beverages, sweets, and desserts. Food group equivalents derived using USDA FPED methodology adapted to Iranian food items. | Krebs-Smith et al., 2018 |
| **Diet Quality Index Revised (DQI-R)** | 10 | Total fat (% energy); saturated fat (% energy); cholesterol (mg/d); fruits (servings/d); vegetables (servings/d); grains (servings/d); calcium (% RDA); iron (% RDA); dietary diversity (food groups); dietary moderation (composite: added sugars, sodium, alcohol, discretionary fat) | Each component scored 0–10 based on cutoff thresholds according to intake quintile or percentage of recommendation. Total fat: ≤30% = 10 pts, >40% = 0 pts. Saturated fat: ≤10% = 10 pts, >13% = 0 pts. Cholesterol: ≤300 mg = 10 pts, >400 mg = 0 pts. Fruit: ≥5 servings = 10 pts. Vegetables: ≥5 servings = 10 pts. Grains: ≥6 servings = 10 pts. Calcium and iron: ≥100% RDA = 10 pts. | 0–100 | Sum of 10 component scores (each 0–10). Higher scores indicate better overall diet quality. Scoring directionality reversed from the original DQI; higher scores reflect better diet quality. | RDA thresholds for calcium (1000 mg) and iron (8 mg for men, 18 mg for women) adapted to standard DRI values. Dietary diversity based on number of unique food groups consumed (out of 23 possible) derived from FFQ items. Dietary moderation score incorporates added sugars, discretionary fat, sodium, and alcohol from FFQ. | Haines et al., 1999 |
| **Energy-Adjusted Dietary Inflammatory Index (E-DII)** | 45 food parameters (original); subset used in this study | **Anti-inflammatory parameters:** MUFA, PUFA, n-3 fatty acids, n-6 fatty acids, fiber, vitamins A, D, E, C, B6, β-carotene, thiamine, riboflavin, niacin, folic acid, magnesium, selenium, zinc, flavan-3-ol, flavones, flavonols, flavonones, anthocyanidins, isoflavones, caffeine, garlic, onion, green/black tea. **Pro-inflammatory parameters:** carbohydrate, protein, total fat, SFA, trans fat, cholesterol, iron, vitamin B12. | Weighted by inflammatory effect score derived from literature review of 1943 articles. For each parameter: Z-score = (individual intake − global mean) / global SD. Z-score converted to percentile, centered (×2, −1), and multiplied by inflammatory effect score. Energy-adjusted to per 1,000 kcal. | Typically −9 to +8 (theoretical) | Sum of all parameter-specific DII scores. Negative values indicate anti-inflammatory diet; positive values indicate pro-inflammatory diet. Energy-adjusted values calculated using the nutrient density method (per 1,000 kcal). | Of 45 DII parameters, 26-28 nutrient parameters available from the Iranian FFQ; flavonoids, isoflavones, garlic, onion, and specific spices not captured and excluded. Global mean and SD values taken from Shivappa et al. 2014 global database (11 datasets from USA, Australia, Bahrain, Denmark, India, Japan, New Zealand, Taiwan, South Korea, Mexico, UK). E-DII scores computed using published SAS macro and validated against the original algorithm. | Shivappa et al., 2014 |
| **DASH Diet Score** | 8 | Fruits; vegetables; nuts & legumes; whole grains; low-fat dairy; sodium; red & processed meats; sugar-sweetened beverages | Quintile-based. Each component scored 1–5 based on sex-specific quintile of intake. Beneficial components (fruits, vegetables, nuts & legumes, whole grains, low-fat dairy): Q1 = 1 point, Q5 = 5 points. Detrimental components (sodium, red & processed meats, sweetened beverages): Q1 = 5 points, Q5 = 1 point. | 8–40 | Sum of 8 component scores. Higher scores indicate greater concordance with DASH dietary pattern. | Sex-specific quintiles derived from study population distribution. Low-fat dairy separated from high-fat dairy using FFQ items for low-fat milk, yogurt, and cheese. Sugar-sweetened beverages include soft drinks and sweetened fruit juices. Sodium estimated from FFQ; discretionary salt use not captured. Component definitions aligned with Fung et al. 2008 methodology. | Fung et al., 2008 |

**Footnotes**

DASH, Dietary Approaches to Stop Hypertension; DQI-R, Diet Quality Index Revised; DRI, Dietary Reference Intake; E-DII, Energy-Adjusted Dietary Inflammatory Index; FFQ, food frequency questionnaire; HEI-2015, Healthy Eating Index 2015; MUFA, monounsaturated fatty acids; PUFA, polyunsaturated fatty acids; RDA, Recommended Dietary Allowance; SFA, saturated fatty acids; USDA, United States Department of Agriculture; FPED, Food Patterns Equivalents Database.
